# Enumerating the gene sets in breast cancer, a "direct" alternative to hierarchical clustering

**DOI:** 10.1186/1471-2164-11-482

**Published:** 2010-08-23

**Authors:** Dwain Mefford, Joel A Mefford

**Affiliations:** 1 Department of Epidemiology and Biostatistics, University of California, San Francisco, San Francisco, California 94107, USA

## Abstract

**Background:**

Two-way hierarchical clustering, with results visualized as heatmaps, has served as the method of choice for exploring structure in large matrices of expression data since the advent of microarrays. While it has delivered important insights, including a typology of breast cancer subtypes, it suffers from instability in the face of gene or sample selection, and an inability to detect small sets that may be dominated by larger sets such as the estrogen-related genes in breast cancer. The rank-based partitioning algorithm introduced in this paper addresses several of these limitations. It delivers results comparable to two-way hierarchical clustering, and much more. Applied systematically across a range of parameter settings, it enumerates all the partition-inducing gene sets in a matrix of expression values.

**Results:**

Applied to four large breast cancer datasets, this alternative exploratory method detects more than thirty sets of co-regulated genes, many of which are conserved across experiments and across platforms. Many of these sets are readily identified in biological terms, e.g., "estrogen", "erbb2", and 8p11-12, and several are clinically significant as prognostic of either increased survival ("adipose", "stromal"...) or diminished survival ("proliferation", "immune/interferon", "histone",...). Of special interest are the sets that effectively factor "immune response" and "stromal signalling".

**Conclusion:**

The gene sets induced by the enumeration include many of the sets reported in the literature. In this regard these inventories confirm and consolidate findings from microarray-based work on breast cancer over the last decade. But, the enumerations also identify gene sets that have not been studied as of yet, some of which are prognostic of survival. The sets induced are robust, biologically meaningful, and serve to reveal a finer structure in existing breast cancer microarrays.

## Background

### Detecting genes-by-samples patterns in expression data

After removing genes that exhibit little variance, the standard script for exploring microarray data applies two-way hierarchical clustering (HC), followed by a visual search for patterns displayed in a red-green heatmap [1,2]. For breast cancer in particular, this procedure has proven immensely productive. It can be credited with the discovery[3] (or rediscovery) [4] of the basal subtype, and, more broadly the identification of subtypes of breast cancer that hold out the potential to inform clinical practice [3,5-8]

Despite its utility, the standard script suffers from several limitations, in particular the instability of the binary tree of the clusters found [9]. Perturbation and re-sampling techniques are available to gauge the robustness of the clusters defined by subtrees [10,11]. But, small changes in the selection of genes or choice of samples can result in disconcertingly large changes in the overall configuration of the tree, which calls into question any typology defined on such tree-based partitions [12].

A different problem stems from the disproportionate impact of large, tightly coordinated clusters on the overall arrangement of the tree[13]. Because the largest gene sets, for example estrogen or immune response, will dominate the branching of the tree, smaller sets may be broken up and redistributed. The problem of large dense sets of genes occluding smaller sets arises in the simple or one-way application of HC; it is compounded when two trees are visually crossed in the two-way HC used in the standard script. The question then becomes one of what can be faithfully represented in the two-dimensional arrangement. The brief answer, as spelled out by Hartigan in the context of "direct" clustering, is that clusters jointly defined by two trees can be rendered as contiguous regions only if they are either disjoint or nested [14,15].

An alternative to two-way hierarchical clustering, biclustering, seeks to find submatrices in the array of expression values that satisfy some defining criteria. Essential to biclustering is the notion that, given a matrix of expression values, the pattern of coordinated expression for a group of genes may be confined to only a subset of samples. That is, the pattern is "local", and may not be detectable using a "global" measure of the similarity between pairs of genes, e.g., Pearson correlation coefficient computed on vectors of expression values [16-18]. Motivated by this concern with detecting "local" structure, several algorithms for detecting biclusters have been advanced. For surveys, see [19,20]. Because of the computational complexity inherent in the biclustering problem, which is provably NP-hard under several formal descriptions[16,18,21], these programs abandon exhaustive search and either resort to heuristics or impose bounds on the size of the genes-by-samples submatrices that can be recovered. The method applied in this paper reframes the biclustering problem such that an exhaustive enumeration of all of the submatrices that meet a certain definition can be achieved. Included in the resulting inventory are not only such familiar sets as "estrogen" and "erbb2/17q12", but additional sets highly prognostic of survival. Some of these newly defined sets may serve alongside "estrogen" etc., as building blocks for future models and classifiers of breast cancer.

## Methods

### Defining gene patterns by partitions on samples

**Figure 1:**
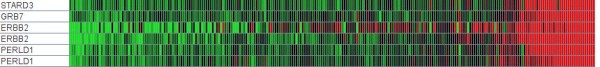
**erbb2/17q12 gene set as detected in the Uppsala data set**. Expression values are mapped to a red-green continuum. Columns are ordered by column sum.

HC is an agglomerative method that recursively joins sets of genes or, separately, sets of samples [22]. The starting point is the notion that similar genes are assigned to the same set (subtree) using a measure of similarity such as correlation[23]. A wholly different approach can be predicated on the notion that two genes belong to the same set provided that they induce the same partition on the set of samples. As an example, the erbb2/17q12 gene set in Figure 1 appears to satisfy such a requirement. Here the samples have been ordered by column sum. It is apparent that the largest (reddest) values of each of these six genes align to a considerable degree. That is, they indicate, or select for, much the same subset of columns (samples). While the notion of genes inducing partitions like this may be intuitive, rendering the notion such that it can be implemented in a search algorithm requires further specifying what constitutes an admissible partition. Crucially important is the partition's size and the degree to which the samples induced by any two genes must align for the partition to be considered the "same". These two parameters (partition "size" and set difference or "tolerance") control a matching rule that can serve to explicitly define what is meant by "gene set".

### Setting up the enumeration

The two parameters that define what counts as the "same partition" suggest a partition-based strategy for finding all sets of genes (all red submatrices) in a matrix of expression values, namely, try all combinations of size and tolerance. Because this collects all the genes-by-samples patterns, it constitutes an *enumeration*.

Since the size and quality of the partitions to be discovered are not known in advance (the procedure is wholly unsupervised), to enumerate all of the partitions in a dataset, and the genes that support them, requires trying all combinations of size and tolerance. This sets up a two dimensional grid search with partition size (*s*) and tolerance (*t*) as dimensions. Given the number of samples (*n*), and the number of draws, that is genes, bounds can be set for the parameters (*s*) and (*t*) by computing p-values with a counting rule (equivalent to the tail of the hypergeometric distribution).

Though the rule for bounding *t *works well enough with smaller datasets, we find that with data sets of the size of the NKI295[24], these p-values are so impossibly small that they provide little guidance in controlling *t*. An alternative strategy relies on a heuristic suggested by results of an initial spiral search in which *s *and *t *are stepped in large increments until gene sets are found, at which point *s *and *t*, are incremented, separately, and in small steps. This preliminary scan of the four datasets finds:

1) For most partition sizes *s*, small (stringent) values of *t *fail to detect any gene sets at all, while large *t *settings yield a single set composed of most, if not all of the genes. Between these two extremes, for any given *s*, there is a relatively narrow range of *t *that returns multiple distinct gene sets.

2) In size and composition, these sets vary smoothly as *s *and *t *are stepped.

3) The number of unique components (gene sets) is surprisingly small, for example, between thirty and forty in the NKI295 data.

In light of these observations from the spiral searches, rather than bounding *t *by a combinatorial counting rule, we choose to set *t *such that specific gene sets remain distinct. In the enumerations reported in this paper, the set we wish to preserve consists of interferon genes, a clinically significant set that the initial spiral search encountered repeatedly. The resulting "stop rule" says, in effect, that for each partition size, which ranges from a small constant (6 or 10), to *n *minus that same constant, increment *t*, starting at 0 (perfect match) until the interferon set disappears as a distinct object as it merges with other immune sets. The motivation for devising this stop rule will be developed further in the context of the gene sets that decompose "immune response".

As the algorithm steps through its search it typically finds multiple versions of the same set, which may differ only marginally in the number and composition of the genes. This means that rather than a single list of genes, a "gene set" in fact refers to a set of sets, like those listed for the ERBB2 set in Table 1. Each column in that table catalogues the genes found for the erbb2/17q12 gene set in the Uppsala data as detected at ten combinations of the "size" and "tolerance" parameters. The list of genes in each column is like a snapshot of the same object in gene space, viewed at a slightly different resolution.

**Table 1:** Instances of the erbb2/17q12 gene set detected in the Uppsala data.

Table 2	**erbb2/17q12 gene set Uppsala data, source = Miller 2005**.								
21t4	23t4	25t5	26t5	27t5	29t6	31t6	32t5	33t6	34t6
ERRB2	ERRB2	ERRB2		ERRB2	ERRB2	ERRB2	ERRB2	ERRB2	
ERRB2	ERRB2	ERRB2	ERRB2	ERRB2	ERRB2	ERRB2	ERRB2	ERRB2	ERRB2
GRB7	GRB7	GRB7	GRB7	GRB7	GRB7	GRB7	GRB7	GRB7	GRB7
PERLD1	PERLD1	PERLD1	PERLD1	PERLD1	PERLD1	PERLD1	PERLD1	PERLD1	PERLD1
PERLD1	PERLD1	PERLD1	PERLD1	PERLD1	PERLD1	PERLD1	PERLD1	PERLD1	PERLD1
STARD3	STARD3	STARD3	STARD3	STARD3	STARD3	STARD3	STARD3	STARD3	STARD3
		CRKRS	CRKRS	CRKRS	CRKRS	CRKRS		CRKRS	CRKRS
		PPARBF	PPARBF	PPARBF	PPARBF	PPARBF		PPARBF	PPARBF
		PPARBF	PPARBF	PPARBF	PPARBF				
		RPL19	RPL19		RPL19				
					CASC3				
					PSMD3				
					THRAP4				
					GSDML				
					GSDML				

The partition size (*s*) and tolerance (*t*) are listed at the head of each column. The first instance of this gene set is detected at *s *= 21, *t *= 4. As *s *and *t *are stepped, the number of erbb2/17q12 genes increases from size to a maximum of 15, at *s *= 29, *t *= 6. From that point the number of genes decreases.

**Figure 2:**
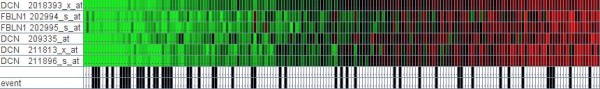
**The survival results for the stromal(5)decorin gene set in the Uppsala cohort**. Columns are ordered by column sum, and recurrence events have been added.

It is important to note that the end product of the enumeration is both the list of genes in each set and the partition induced on the samples by that set. While the number of genes in the sets can vary from six (the lower bound) to as many as 600 (as in the case of the proliferation gene set), regardless of the number of genes in the set there can be only one partitioning pattern per gene set. It is in this context that the issue of genes with multiple probe sets can be addressed. As would be expected, multiple probe sets spotted for the same gene will, to the extent that they induce the same partition on the samples, be assigned to the same gene set by the algorithm. A case in point is the small stromal gene set in Figure 2 which consists principally of decorin (DCN) and fibulin 1 (FBLN1). Decorin is spotted four times on the Affymetrix HG-U133a and the algorithm finds that all four decorin probe sets induce the same partition pattern on the 251 samples in the Miller dataset [25]. That same pattern is also induced by two copies of FBLN1, as well as by single instances of GLT8D2, CTSK, PRRX1, and SPON1, and several ESTs. All in all, in the Miller data, this partitioning pattern is found at five combinations of *s *and *t *settings. For the purpose of making the survival analysis manageable, we summarize (squash) these five versions of this gene set into one composed of those probe sets that appear in at least half of the original versions. The result is the set pictured in the heatmap in the figure, to which metastasis events have been attached. As described more fully in the Results section, the χ^2 ^for a partition at the median value of a vector of column sums is 7.62 (p = 0.005). The χ^2 ^for first versus last quartile is 15.62 (p = 0.00007). Since each of the genes in a gene set realize the same partitioning pattern, the end result, in terms of partitioning and survival analysis would be virtually the same if the four decorin probe sets were replaced by any one (or if the four were averaged). This issue of multiple probe sets for the same gene does become a problem for the detection of the smallest gene sets in the case in which the partitioning pattern is realized exclusively in probe sets all of which spot the same gene. This occurs occasionally in the Affymetrix data, and we consider these instances to be artefacts of the chip design.

### Detecting patterns that were heretofor undetectable

The virtues of HC and the standard script include the fact that it is intuitive, simple to implement, and effective (at least in finding the largest and most prominent genes-by-samples patterns). Moreover, the software that implements this method of discovery is free and well maintained [26,27], and the output is not only immanently useful, but a thing of beauty. Replacing this standard script requires both a conceptual shift and considerable computational apparatus. For these reasons, such a switch can only be justified if the alternative not only reproduces the standard results, but significantly extends those results. That is, it must find additional patterns that are biologically meaningful and clinically significant. To show that this is in fact the case we enumerate the gene sets in four well-studied breast cancer microarray data sets listed in Table 2. We refer to these as: Uppsala, Stockholm, TRANSBIG, and NKI data sets. Three of these use Affymetrix U133a chips, a platform that has attained the status of an industry standard, and has been used perhaps in more studies than any other microarray platform. To identify gene sets conserved across platforms, we add the fourth data set, generated by the Nederlands Kanker Instituut (NKI), which used a custom Agilent chip. The Uppsala and Stockholm cohorts [25,28] are population studies (consecutively presented primary breast cancers). The TRANSBIG dataset is a validation series for a gene-based classifier [29] and, accordingly, consists of younger (median age 47) node-negative patients [30]. The NKI data was collected in conjunction with a second classifier [24], and consists of younger patients, both node-positive and node-negative. For purpose of survival analysis in this paper, the end point for the Uppsala data is Breast Cancer Specific Survival (BCSS); the end points in the other three data sets are Distant Metastasis Free Survival (DMFS). The datasets were selected, in the first instance, because of the quality of the data, both expression and accompanying clinical data. But they were also chosen to set up explicit comparisons between the gene sets detected by the enumerations in this paper and gene clusters and signatures induced on the same data by other methods and programs.

**Table 2:** Data sets.

dataset	source	n	survival endpoint	median/average follow-up	reference
Uppsala	GSE3494	251	BCSS	10.2 years	Miller 2005
Stockholm	GSE1456	159	DMFS	6.1 years	Pawitan 2005
TRANSBIG	GSE7390	198	DMFS	13.6 years	Desmedt 2007
NKI	Chang	295	DMFS	6.7 years	VandeVijver 2002

Data sets were downloaded from the GEO website [31], or, in the case of the NKI data, from the Stanford website [32] that accompanies [33,34]. Missing values were estimated using k Nearest Neighbours, (k = 10) [35]. Because the algorithm takes the full expression matrix as input, e.g., in the case of the Uppsala data, a matrix of size 22,283 × 251, there is no prior filtering/selection of genes.

## Results

In each of four breast cancer datasets in Table 2, the rank-based bi-clustering algorithm finds between thirty and forty sets of genes. Many of these sets are conserved across the four studies despite differences in platform: Affymetrix U133a (Uppsala, Stockholm, TRANSBIG), and custom Agilent (NKI). Several of the largest and most prominent of the sets closely resemble gene clusters reported many times in microarray studies of breast cancer. These include "estrogen", "proliferation", ERBB2, and "adipose" [1,2,9,36-38]. Other sets, a number of which are relatively small (e.g., 6 to 12 genes), appear to be novel. These include "histone", "hemoglobin" "amplicon 8p11-12". Overall, nearly a third of the gene sets detected are clinically significant as measured by association with survival, and some are very significant with a log-rank χ^2 ^values exceeding 20 (on one degree of freedom).

For purpose of presentation, in the course of enumerating the gene sets in each of the four data sets, we proceed in three steps. The gene sets detected in each of the three Affymetrix data sets are first tabulated separately. This is followed by a comparison of these sets to identify gene sets conserved on the Affymetrix U133a platform. This, then, is followed by an enumeration of the gene sets in the NKI295 data set which uses a custom Agilent chip. These sets are then compared to the Affymetrix sets for the purpose of identifying gene sets conserved across the two platforms.

Regarding the clinical significance of the gene sets, since a gene set, in general, is detected multiple times (resulting in multiple gene lists), the survival analysis of a gene set would yield multiple log-rank values. To simplify the analysis we have replaced these multiple versions of each set with a "core" set comprised of those genes that appear in at least half of the instances detected. Five log-rank χ^2 ^values are reported for each core gene set. The first reflects all the samples in a data set partitioned at the median value of the column sums of the genes in the set. The second partitions all samples by lowest and highest quartiles. The third restricts the samples to those which are ER positive, and partitions at the median. The fourth also restricts the samples to ER positive, partitioning by quartiles. The fifth restricts the samples to ER negative, partitioned at the median. Because of small class size, the ER negative samples are not partitioned by first and last quartile.

### Dataset 1: Uppsala cohort (Miller 2005)

Table 3 reports the survival analysis for the 35 gene sets induced in the Uppsala data. The rows have been arranged with gene sets associated with increased breast cancer specific survival are at the top, and the sets prognostic of diminished survival are at the bottom. A list of the genes in the top ten sets and bottom seven sets are provided in Additional File 1. A complete list of genes for all 35 sets is available in Additional File 2. The most striking result is the large, positive significance of the four stromal sets, which are discussed in a later section. Also predictive of increased survival are two immune sets, and estrogen and adipose, as might be expected.

**Table 3:** Gene sets and survival results for the Uppsala data.

	all samples				ER positive				ER negative	
	median		quartiles		median		quartiles		median	
	**χ**^**2**^	p	**χ**^**2**^	p	**χ**^**2**^	p	**χ**^**2**^	p	**χ**^**2**^	p
stromal(0)	**6.51**	**0.01**	**6.54**	**0.01**	**4.07**	**0.04**	**5.3**	**0.02**	**3.95**	**0.04**
stromal(2)	**13.94**	**0.0001**	**10.76**	**0.001**	**10.17**	**0.001**	**10.74**	**0.001**	1.16	0.28
stromal(3)	**6.43**	**0.01**	**8.05**	**0.004**	**7.59**	**0.005**	**7.72**	**0.005**	**4.32**	**0.03**
stromal(5)	**7.62**	**0.005**	**15.62**	**7E-05**	**6.66**	**0.009**	**12.4**	**0.0004**	3.22	0.07
adipose	**6.96**	**0.008**	**12.72**	**0.0003**	**5**	**0.02**	**8.96**	**0.002**	0.99	0.31
immune(5)	**4.48**	**0.034**	**5.06**	**0.02**	2.84	0.09	2.95	0.08	2.64	0.1
immune(6)	**9.63**	**0.001**	**7.19**	**0.007**	**5.78**	**0.01**	**4.29**	**0.03**	3.41	0.06
immune(0)	1.85	0.17	0.07	0.79	0.9	0.34	0.02	0.89	**4.26**	**0.03**
TPSAB1	2.92	0.08	**4.44**	**0.03**	**5.39**	**0.02**	2.81	0.09	0.95	0.32
estrogen	**4.36**	**0.03**	0.65	0.42	2.36	0.12	1.15	0.28	0.5	0.48
										
AFFX-BioC-5	0	0.95	0.4	0.52	0.16	0.68	0.49	0.48	0.59	0.44
ACTG1	0.12	0.72	0	0.96	0.7	0.4	0.06	0.8	0.74	0.38
basal	0.94	0.33	1.13	0.28	0.98	0.32	2.32	0.12	0.37	0.54
ERBB2	2.83	0.09	3	0.08	2.27	0.13	2.4	0.12	0	0.97
GGT1	0	0.99	0	0.96	0.05	0.81	0.1	0.74	0	0.99
hemoglobin	0.03	0.86	0.06	0.81	0.32	0.56	0.55	0.45	0	0.94
immune(1)	1.54	0.21	0.05	0.82	1.07	0.3	0.38	0.53	3.32	0.06
immune(2)	0.06	0.8	0.44	0.5	0.18	0.67	0.24	0.62	0.46	0.49
immune(3)	1.71	0.19	1.98	0.15	1.61	0.2	1.26	0.26	1.15	0.28
immune(4)	2.21	0.13	0.68	0.4	1.76	0.18	1.27	0.25	0	0.99
immune(7)	0.5	0.47	0.1	0.74	0.93	0.33	0.01	0.93	0.79	0.37
LST1	3.25	0.07	0.82	0.36	1.69	0.19	0.25	0.61	1.04	0.3
NFIB	0.55	0.45	0.03	0.85	0.02	0.87	0.01	0.91	0.22	0.63
PPP1R12A	0.01	0.94	1.19	0.27	0	0.95	1.34	0.24	0.08	0.77
ribosomal(0)	0.35	0.55	1.7	0.19	0.36	0.55	0.53	0.46	0	0.96
ribosomal(1)	0.63	0.42	0.07	0.79	0.14	0.7	0.03	0.87	0.03	0.86
ribosomal(5)	0.19	0.66	0.51	0.47	0.22	0.64	0.12	0.73	0.97	0.32
UBE2D2	0	0.99	0.43	0.51	0	0.99	0.78	0.37	0.13	0.72
										
histone	**4.65**	**0.03**	0.78	0.37	3.44	0.06	1.22	0.27	3.11	0.07
GAPDH	3.07	0.07	**4.73**	**0.02**	2.35	0.12	2.09	0.14	3.7	0.05
CD24	0.45	0.5	5.25	0.02	0.82	0.36	**4.47**	**0.03**	0.51	0.47
AFFX-M27830_5	1.68	0.19	4.3	0.03	1.72	0.19	**6.27**	**0.01**	0.01	0.92
GNAS	3.34	0.06	**6.15**	**0.01**	2.07	0.15	3.04	0.08	1.36	0.24
16q13	2.58	0.1	**5.33**	**0.02**	2.49	0.11	**8.07**	**0.0004**	2.4	0.12
proliferation	**10.36**	**0.001**	**14.59**	**0.0001**	**6.78**	**0.009**	**13.22**	**0.0002**	0.71	0.39

The proliferation set is strongly prognostic of recurrence in the Uppsala data, as it is in the other three data sets, which accords with a number of microarray-based studies of breast cancer [39,40]. Other sets strongly prognostic of recurrence are the histones, the metallothioneins/16q13, as well as GAPDH, CD24, AFFX-M27830_5, and GNAS. The histone set includes *HIST1H2BF HIST1H2BE HIST1H2BH H2BFS HIST1H2BK HIST1H2BD*, while the metallothioneins gene set consists of seven, or perhaps eight, isoforms and two ESTs: *MT1E, MT1F, MT1G, MT1 H, MT1M*, MT1X, *MT2A, LOC645745*. Up-regulation of *MT2A*, the most abundant of these, is associated with more aggressive breast cancer and poor prognosis [41], and it is reported that the over-expression of metallothioneins predicts resistance to doxorubicin [42].

*GNAS *is located on the long arm of chromosome 20 at 20q13.3, lying just outside the interval of the 20q13 amplicon investigated by Ginestier et al.[43], but falling within one of the two 20q13 amplicons analyzed by Yao et al [44]. It is reported that increased copy numbers for 20q13 amplicon genes occur in 12% of primary breast tumors, and are associated with more aggressive disease [45].

*GAPDH *is often used as a housekeeping gene [46]. For example it is one of the five genes used to normalize recurrence score in OncogeneDX [47,48]. But, *GAPDH *is reported to be up-regulated in some cancers, e.g., by a factor of 3 to 6 in non small cell lung cancer compared to normal lung tissue [49]. In a study of *GAPDH *expression in breast cancer, Revillion et al.,[50] conclude with the warning that it should not be used as a control RNA. Valenti et al reach a similar conclusion [51]. The fact that the *GAPDH *partitions the Uppsala samples into good and poor prognostic groups is added evidence that it is not only unsuitable for scaling expression values, but may in fact be a candidate oncogene.

### Data set 2: Stockholm cohort (Pawitan 2005)

Similar to the results for the Uppsala data, for the Stockholm cohort three stromal gene sets are strongly associated with increased survival as reported in Table 4. Also, again, the adipose set proves significant. In addition, among the sets associated with increased survival is a hemoglobin set consisting of: *HBH1, HBB*, *HBA1, HBB, HBA1, HBA2, HBA2, HBB, HBA2, HBG1 *(where the order of the genes reflect the row order of the probe sets on the Affymetrix U133a). A complete list of the genes in all 31 gene sets for the Stockholm cohort is available in Additional File 3. Though ER and PR status are reported in the aggregate in the original article[52], ER status was not available for this data set. Consequently the log-rank values tabulated in Table 4 represent all 159 samples partitioned at the median, and by first and last quartiles. Among the sets significantly associated with decreased survival, as in the Uppsala data, proliferation is most prominent. Also, again, the histone set, CD24, and GAPDH figure among the sets associated with recurrence. In addition, among the gene sets that have a negative impact on survival in this data set include ACTG1, a ribosomal set, and a set we have labelled ezrin, because it includes the gene *VIL2*. Ezrin expression is associated with metastasis in a number of cancers [53-55]. In a murine cell line Elliot et al show that ezrin is required for metastasis [56], and Li et al demonstrated that ezrin silencing reverses cell migration and invasion in a metastatic breast cancer cell line [57]. With regard to survival in breast cancer, Bruce et al report that ezrin expression is associated with poor outcome [58]. The ezrin gene set also contains *ARF1*, which is reported to modulate migration and proliferation in breast cancer cell lines via the regulation of the PI3K pathway [59].

**Table 4:** Gene sets and survival results for the Stockholm data.

	all 159 samples			
	median		quartiles	
	**χ**^**2**^	p	**χ**^**2**^	p
stromal(2)	**20.01**	**0.000007**	**11.64**	**0.0006**
stromal(3)	**13.52**	**0.0002**	**11.58**	**0.0006**
stromal(5)	**13.79**	**0.0002**	**12.72**	**0.0003**
adipose	**4.66**	**0.03**	**4.13**	**0.04**
hemoglobin	3.33	0.06	**4.4**	**0.03**
				
16q13	2.62	0	2.12	0.14
AFFX-BioC-5	0.03	0.86	0.26	0.6
AFFX-M27830_5	0.15	0.69	2.09	0.14
basal	3.42	0.06	1.14	0.28
estrogen	0.02	0.88	0.98	0.32
GGT1	1.61	0.2	0.76	0.38
stromal(0)	2.49	0.11	2.35	0.12
stromal(1)	0.98	0.32	0.08	0.78
immune(0)	0.13	0.72	0.1	0.75
immune(1)	0	0.96	0.35	0.55
immune(2)	1.1	0.29	1.11	0.29
immune(5)	1.25	0.26	0	0.96
LST1	0.43	0.51	0.02	0.88
OPHN1	0.04	0.84	0.02	0.89
PPP1R12A	2.16	0.14	3.11	0.07
ribosomal(0)	0.72	0.39	0.63	0.42
TPSAB1	0.22	0.64	0.49	0.48
UBE2D2	0	0.99	1.9	0.16
				
immune(4)	3.36	0.06	**3.67**	**0.05**
ezrin	3.35	0.06	**3.7**	**0.05**
ACTG1	**3.85**	**0.04**	1.65	0.19
ribosomal(1)	**3.64**	**0.05**	**5.45**	**0.19**
histone	**6.76**	**0.009**	**5.98**	**0.01**
CD24	2.7	0.1	**8.9**	**0.002**
GAPDH	**10.96**	**0.0009**	**8.76**	**0.003**
proliferation	**19.88**	**0.000008**	**13.09**	**0.0002**

### Data set 3: TRANSBIG (Desmedt 2007)

While the TRANSBIG data employs the same Affymetrix platform as the Uppsala and Stockholm datasets, it differs in terms of sample selection. The first two data sets are population-based, while this data set, as a validation series, reflects the sampling criteria used in the development of Wang et al.'s 76-gene classifier [29]. As a consequence, patients were younger (less than 61 years of age, with a median age of 47), node-negative, with smaller tumour grade (T1-T2, less than 5 cm). Despite these differences, as is apparent in Table 5, the gene sets detected are largely the same as those found in the Uppsula and Stockholm cohorts. A complete list of the genes in each of the 37 sets discovered in the TRANSBIG data are available in Additional File 4. As in the previous two data sets, stromal(2) is associated with increased survival. Distinctive of this third data set are the three ribosomal sets associated with survival, a relationship which for two of the sets (ribosomal(0) and ribosomal(3)) is particularly strong for ER positive samples.

**Table 5:** Gene sets and survival results for the TRANSBIG data.

	all 198 samples				ER positive				ER negative	
	median		quartiles		median		quartiles		median	
	**χ**^**2**^	p	**χ**^**2**^	p	**χ**^**2**^	p	**χ**^**2**^	p	**χ**^**2**^	p
ribosomal(0)	**4.04**	**0.04**	**8.21**	**0.004**	**9.07**	**0.002**	**10.38**	**0.001**	0.06	0.8
ribosomal(3)	3.38	0.06	**5.51**	**0.01**	**8.93**	**0.002**	**12.91**	**0.0003**	0.46	0.49
ribosomal(4)	1.94	0.16	**4.08**	**0.04**	3.18	0.07	**3.67**	**0.05**	0.02	0.88
stromal(2)	**3.57**	**0.05**	1.54	0.21	2.45	0.11	0.44	0.5	0.22	0.63
AFFX-BioC-5	1.64	0.2	0.47	0.49	**3.56**	**0.05**	0.1	0.75	2.75	0.09
										
adipose	1.67	0.19	1.1	0.29	3.31	0.06	1.17	0.27	0	0.96
ACTG1	2.53	0.11	0.43	0.51	0.31	0.57	0	0.98	0.49	0.48
CD24	0.17	0.67	2.84	0.09	0.45	0.5	0.81	0.36	0.13	0.72
CD44	0.31	0.57	0.05	0.81	0.41	0.52	0.05	0.86	0.01	0.92
CFLAR	1.66	0.19	0	0.99	0.82	0.36	2.84	0.09	1.04	0.3
ERBB2	0.75	0.38	0.01	0.93	0.94	0.33	2.75	0.09	0.77	0.38
estrogen	0.24	0.62	0.57	0.44	0.28	0.59	2.11	0.14	0.54	0.46
FOXA1	1.1	0.29	0.27	0.6	0.29	0.58	0.19	0.66	0.58	0.44
GAPDH	0.35	0.55	2.03	0.15	2.05	0.15	0.18	0.67	0.01	0.9
GGT1	0.87	0.35	0	0.99	0.03	0.86	0.04	0.84	1.52	0.21
hemoglobin	0.24	0.62	0.53	0.46	0.31	0.57	0.11	0.73	0.74	0.38
immune(0)	0.05	0.81	0.57	0.44	0.22	0.64	0	0.96	1.35	0.24
immune(1)	1.01	0.31	0.56	0.45	0.87	0.34	0.94	0.33	2.2	0.13
immune(2)	0.31	0.57	0.17	0.68	0.49	0.48	0.1	0.74	0.01	0.94
immune(4)	0.04	0.83	0.01	0.92	0.05	0.81	0	0.98	2.11	0.14
immune(5)	1.26	0.26	0.04	0.84	0.68	0.4	1.27	0.26	0.23	0.63
immune(6)	0.08	0.77	0.1	0.75	0.19	0.66	0.67	0.41	0.97	0.32
immune(10)	1.81	0.17	0.47	0.49	1.71	0.19	1.97	0.16	0.97	0.32
myo	0.22	0.63	0.39	0.53	0.11	0.73	0	0.94	3.01	0.08
NFIB	0.56	0.45	0.12	0.73	0.41	0.52	2.12	0.14	0	0.98
PPP1R12A	1.47	0.22	0.26	0.61	0.51	0.47	1.26	0.26	0.63	0.42
SMARCA4	0.08	0.77	0.43	0.51	1.04	0.3	2.79	0.09	1.99	0.15
stromal(0)	0.29	0.59	2.37	0.12	0.41	0.52	1.6	0.2	0.01	0.92
stromal(1)	2.51	0.11	1.46	0.22	2.21	0.13	0.08	0.77	2.36	0.12
stromal(5)	0.58	0.44	0.25	0.61	0.03	0.85	0.8	0.37	1.69	0.19
TCF4	0.87	0.35	0.13	0.71	0.94	0.33	0.55	0.45	0.92	0.33
TPSAB1	0.21	0.64	0.03	0.86	0.23	0.63	0.29	0.59	0.03	0.86
UBE2D2	0.05	0.81	0	0.95	1.07	0.3	2.82	0.09	0.42	0.51
										
basal	0.13	0.71	2.48	0.11	**4.52**	**0.03**	2.32	0.12	0.35	0.55
histone	1.54	0.21	**4.04**	**0.04**	0.26	0.61	2.21	0.13	0.33	0.56
NKTR	1.29	0.25	0.03	0.85	**4.41**	**0.03**	1.55	0.21	1.54	0.21
16q13	0.86	0.35	0.23	0.63	0.4	0.52	0.27	0.6	**5.28**	**0.02**
proliferation	**5.27**	**0.02**	**3.69**	**0.05**	**9.36**	**0.002**	**5.8**	**0.01**	**3.89**	**0.04**

With regard to gene sets prognostic of decreased survival, again, as with the Uppsala and Stockholm cohorts, histone and proliferation figure prominently, as do the metallothioneins. Also among the sets associated with decreased survival are the basal set and NKTR.

### Comparing gene sets across the three Affymetrix U133a data sets

Factors that impact the ability of the algorithm to detect sets that induce a common partition include the design of the microarray platform. Some genes, for example *CD24 *or *LST1*, are rendered by multiple probe sets. To the extent that they take similar expression values for the same samples, the algorithm will identify them as a (small) gene set in their own right. That is, all gene sets that satisfy the matching criteria are reported in the enumeration, including several as small as six genes in size. While some of these will prove to be artefacts of chip design, others may prove to be significant, for example the eight-gene 8p11-12 gene set detected in the NKI data. A virtue of the method over HC is this capacity to find even the smallest sets that induce important patterns on the samples.

Table 6 juxtaposes the sets found in each of the three Affymetrix data sets (Uppsala, Stockholm, and TRANSBIG). As apparent from the table, there is a remarkable concordance: to a considerable extent the same thirty, or so, sets are extracted from independently assembled matrices of expression values, each of which is comprised of as many as five million real numbers.

**Table 6:** Gene sets detected in the three Affymetrix U133a data sets.

Uppsala	TRANSBIG	Stockholm
adipose	adipose	adipose
basal	basal	basal
estrogen	estrogen	estrogen
proliferation	proliferation	proliferation
erbb2	erbb2	
immune(0)	immune(0)	immune(0)
immune(1)	immune(1)	immune(1)
immune(2)	immune(2)	immune(2)
immune(3)		
immune(4)	immune(4)	immune(4)
immune(5)	immune(5)	immune(5)
immune(6)	immune(6)	
immune(7)		
	immune(10)	
stromal(0)	stromal(0)	stromal(0)
	stromal(1)	stromal(1)
stromal(2)	stromal(2)	stromal(2)
stromal(3)		stromal(3)
stromal(5)	stromal(5)	stromal(5)
ribosomal(0)	ribosomal(0)	ribosomal(0)
ribosomal(1)		ribosomal(1)
	ribosomal(3)	
	ribosomal(4)	
ribosomal(5)		
metallothioneins	metallothioneins	metallothioneins
ACTG1	ACTG1	ACTG1
AFFX-BioC-5_at	AFFX-BioC-5_at	AFFX-BioC-5_at
CD24	CD24	CD24
	CD44	
GAPDH	GAPDH	GAPDH
GGT1	GGT1	GGT1
hemoglobin	hemoglobin	hemoglobin
histone	histone	histone
PPP1R12A	PPP1R12A	PPP1R12A
TPSAB1	TPSAB1	TPSAB1
UBE2D2	UBE2D2	UBE2D2
NFIB	NFIB	
AFFX-M27830_5		AFFX-M27830_5
LST1		LST1
GNAS		
	NKTR	
	FOXA1	
	myo	
	CFLAR	
	SMARCA4	
	TCF4	
		ezrin
		OPHN1

### Data set 4: NKI 2002

Of the 35 gene sets discovered in the enumeration of the NKI295, available in Additional File 5, eight are significantly associated with increased survival. Of these, the two largest (in terms of number of genes) are the estrogen set, well-documented in microarray-based studies of breast cancer [60], and a second set that we have labelled "FOXA1". These sets are disjoint except for the pivotal *FOXA1 *(*HNF3A*) gene which appears in both, and which is represented by two probes on the custom Agilent chip used in the NKI data. While the estrogen and FOXA1 gene sets are both significantly associated with increased survival, the two sets induce substantially different partitions on the 295 tumor samples, possibly suggesting different positive mechanisms at work.

The gene *FOXA1 *is known to correlate strongly with estrogen receptor alpha [61-63], and the mechanism that accounts for this association has been established, namely, ER binding requires *FOXA1 *binding in close proximity [64,65]. But, it has also been shown that *FOXA1 *expression is largely independent of estrogen receptor status. A substantial proportion of ER-negative tumors express *FOXA1 *[66], while, in one study, more than 40% of ER-positive tumors were down-regulated for *FOXA1 *[67]. Additional support for the notion that *FOXA1 *is independent of ER, is the fact that most of the known *FOXA1*-response genes are not ERα-response genes [67,68]. So, paradoxically, it would appear that *FOXA1 *is both correlated and uncorrelated with ER. The estrogen and FOXA1 sets detected in the enumeration, support both propositions. It should be noted that at least two other genes in the FOXA1 set, *TLOC1 *and *SDCCAG1*, are reported to act as tumor suppressors in their own right [69,70].

Two stromal sets, described in the next section, are shown in Table 7 to be associated with increased survival.

**Table 7:** Gene sets and survival results for the NKI data.

35 gene sets	all 295 samples				ER positive				ER negative	
	median		quartiles		median		quartiles		median	
	**χ**^**2**^	p	**χ**^**2**^	p	**χ**^**2**^	p	**χ**^**2**^	p	**χ**^**2**^	p
estrogen	**11.33**	**0.0007**	**9.29**	**0.002**	**7.91**	**0.004**	**5**	**0.02**	2.9	0.08
FOXA1	**4.4**	**0.03**	**3.62**	**0.05**	**7.82**	**0.005**	1.72	0.18	0.09	0.76
stromal(5)	2.73	0.09	2.13	0.14	**4.03**	**0.04**	3.21	0.07	0.02	0.88
stromal(4)	**3.8**	**0.05**	**8.63**	**0.003**	**7.34**	**0.006**	**6.13**	**0.01**	0.01	0.9
MAGEB1	**5.77**	**0.01**	0.82	0.36	1.83	0.17	0.19	0.66	0.41	0.52
8p11_12	2.56	0.1	**4.2**	**0.04**	**5.05**	**0.02**	**6.39**	**0.01**	0.4	0.52
immune(0)	0.01	0.92	0.11	0.74	2.8	0.09	1.25	0.26	**8.21**	**0.004**
immune(1)	0.81	0.36	0.07	0.78	0.03	0.86	0.82	0.36	**4.5**	**0.03**
										
AANAT	0.8	0.77	0.03	0.85	0.03	0.58	0.72	0.39	2.32	0.12
ACACB	0.03	0.85	0.85	0.35	1.31	0.25	0.67	0.41	1.38	0.24
adipose	0.13	0.71	0.69	0.4	0.66	0.41	0.21	0.64	0.01	0.92
basal	0.16	0.68	0.4	0.52	0.01	0.93	0.11	0.73	0.4	0.52
BCL2L1	0.59	0.44	0.4	0.52	3.97	0.04	1.22	0.26	0.01	0.92
EMX2	0.75	0.38	1	0.31	0.25	0.61	0.04	0.84	0.48	0.48
ERBB2	0.34	0.55	0.55	0.45	0.76	0.38	0.25	0.61	0.08	0.77
H3_histone	0.27	0.6	0.17	0.68	0.27	0.6	0.11	0.74	0.02	0.89
immune(5)	0.8	0.37	0.06	0.8	3.01	0.08	2.23	0.13	0.98	0.32
immune(9)	0.34	0.55	0	0.99	0.85	0.35	0	0.94	0	0.97
JAG2	0.12	0.72	0.14	0.71	0.17	0.68	0.22	0.63	0.01	0.9
KRT1	1.7	0.19	0.81	0.36	2.58	0.1	3.21	0.07	0.71	0.4
MMP17	0.02	0.89	1.42	0.23	0	0.95	0.11	0.74	0.65	0.42
NRGN	0.06	0.8	0.87	0.35	0.09	0.76	0.43	0.51	0.35	0.55
RAB18	0.34	0.55	1.77	0.18	0.57	0.44	0.15	0.69	0.06	0.8
RAPGEF2	1.25	0.26	1.23	0.26	1.46	0.22	0.19	0.66	2.48	0.11
RBM5	0.03	0.85	0.65	0.42	0.24	0.62	0.85	0.35	0.31	0.57
ribosomal(0)	0.01	0.93	0.37	0.54	0.09	0.77	0.14	0.71	0.12	0.73
ribosomal(2)	0.8	0.36	1.48	0.22	0.52	0.47	1.19	0.27	0.21	0.64
SEMG1	2.67	0.1	2.25	0.13	2.3	0.12	2.06	0.15	2.99	0.08
stromal(0)	0.31	0.57	0.62	0.42	2.41	0.12	2.33	0.12	0.28	0.59
										
PTPRO	0.02	0.89	2.84	0.09	0.22	0.63	**4.53**	**0.03**	0	0.97
ATP12A	**4.45**	**0.03**	3.04	0.08	1.78	0.18	1.49	0.22	**5.35**	**0.02**
immune(2)	**4.61**	**0.03**	2.68	0.1	**7.76**	**0.005**	**6.42**	**0.01**	0.4	0.52
immune(3)	**5.71**	**0.01**	**3.97**	**0.04**	**8.72**	**0.003**	**9.78**	**0.001**	1.01	0.31
H2B_histone	**6.79**	**0.009**	**3.64**	**0.05**	**9.96**	**0.001**	**6.11**	**0.01**	0.3	0.58
proliferation	**24.79**	**6E-07**	**29.09**	**6E-08**	**22.98**	**1E-06**	**25.73**	**3E-07**	**4.15**	**0.04**

The genes in the 8p11-12 gene set essentially tile a region of 8p11-12 amplicon between 37 M and 38.5 M. The set largely agrees with the amplicon as refined by Haverty et al. [71]. Seven of the eight genes map to one of the four 8p11-12 sub-amplicons that Gelsi-Boyer et al identified using array-CGH and Reyal et al.'s method for correlating genes within 20-gene windows [72,73]. Studies have found that the expression of each of these genes is significantly correlated with copy number [74,75].

It is interesting to observe that immune sets are associated with survival both positively (immune(0)T-cell and immune(1)IgG) and negatively (immune(2)MHC-I and immune(3)interferon). As with the three Affymetrix data sets, the histone and proliferation gene sets are strongly prognostic of poor outcome in the NKI data. Two histone sets, H2B_histone and H3_histone are detected in this data set, but it is only the H2B, which corresponds to the histone set in the Affymetrix data, that is significant.

### Gene sets conserved across the Agilent and Affymetrix platforms

**Figure 3:**
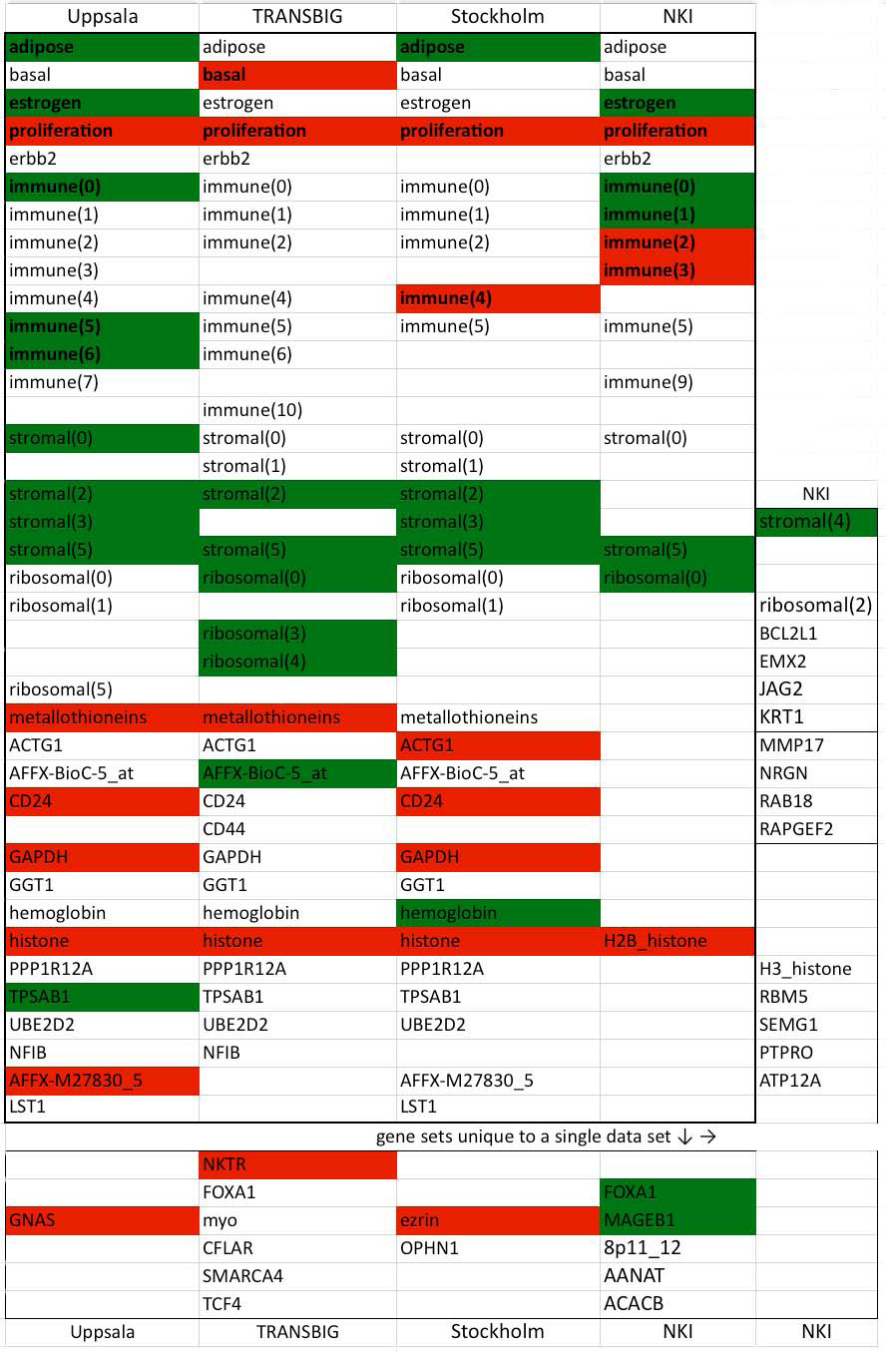
**Gene sets detected across Affymetrix and Agilent platforms**. Red indicates significant association with decreased survival; green indicates significant association with increased survival.

Figure 3 attempts to present an overview of all the sets induced across all four data sets. The inner box contains the sets found in two or more of the data sets; the outer box lists sets unique to individual data sets. Sets significantly associated with increased survival are marked in green; sets associated with decreased survival are marked in red. It is apparent that the proliferation and histone sets are robust indicators of poor prognosis, while several of the stromal and ribosomal sets are associated with better outcomes, possibly conferring some form of protection against recurrence. The immune sets are a mixed bag, pointing in both directions.

## Discussion

While the tables in the Results Section report the gene sets found and their clinical significance, here the purpose is to compare gene sets, in particular those related to immune response and stromal signalling, to gene clusters and signatures identified across a number of microarray studies. For many of the sets detected the correspondence is immediate. This is the case in particular for "estrogen", "erbb2", "basal", and "proliferation", all of which have become virtual fixtures in genomic studies of breast cancer [2,39,76]. Other sets, including several small sets found only in the Affymetrix data, may be trivial in the sense that they consist of housekeeping genes, or reflect the design decision to spot single genes multiple times. In these cases, the resulting patterns, as detected by the algorithm, are real, but may be of only technical interest in so far as they concern issues of normalization and quality control. Of the remaining sets detected by the enumerations, several are easily described and readily interpreted in biological terms. Examples include:

• hemoglobin: *HBA1, HBB, HBA2, HBG1*...

• histone: *HIST1H2BF, HIST1H2BE, HIST1H2BFS, H2BFS, HIST1H2BK*, ...

• metallotheioneins: *MT1G, MT1 H, MT1X, MT2A, MT1F, MT1E, MT1M*

Of special interest are the ten sets labelled "immune", and the six or more identified as "stromal".

### A natural factoring of Immune Response

Eight immune sets are detected in the Uppsala data, five in the Stockholm, seven in TRANSBIG, and six in NKI. All told, across the four data sets, ten unique immune sets are induced. Of these, two are questionable: one consisting essentially of five copies of *CASP1*, and the other, a small set in the NKI containing *TNFRSF17*, which is most likely a subset of the gene set labelled "immune(1)IgG". Setting these aside, the broad category of "immune-related genes" appears to factor naturally into eight distinct subcategories. To marshal evidence for, and against, this division, and to aid in their biological interpretation, these eight sets can be matched against clusters and lists of immune genes identified by others using standard methods, generally hierarchical clustering. The correspondence is one-one between the immune gene sets and eight of Loi's et al's "pclusts" [77] and seven of Rody et al.'s immune-related metagenes [78]. Examining this correspondence in greater detail, seven of the eight "immune" sets correspond almost exactly to the gene clusters used by Rody et al. to compute immune "metagenes" in their study of lymphocytic infiltrates in breast cancer [78]. In that study of twelve Affymetrix breast cancer data sets, hierarchical clustering repeatedly yielded a cluster of approximately 600 immune-related genes. Applying hierarchical clustering a second time to this cluster revealed seven distinct sub-clusters. The concordance between the genes in these seven clusters and seven of the immune sets as detected in the enumeration of the Uppsala data is available in Additional File 6. The one minor discrepancy between the enumerated sets and these seven clusters involves the five *LST1 *probe sets in the monocyte cluster (last column). In the enumerations of the Uppsala and Stockholm cohorts, *LST1 *forms its own small gene set.

The survival analysis on the two largest datasets, Uppsala and NKI, confirm Rody et al.'s principal finding [78], namely T-cell genes are prognostic of increased survival time for estrogen-negative patients, with χ^2 ^= 4.26, p = 0.03 for the Miller data, and χ^2 ^= 8.21, p = 0.004 for the Van de Vijver data. But on these same datasets, contrary to Rody et al. who find no association between B-cell/immunoglobulin genes and survival, immune(1)IgG is a significant predictor of increased survival in the NKI data, χ^2 ^= 4.5, p = 0.03, and marginally so in the Uppsala data, χ^2 ^= 3.32, p = 0.06. The genes in immune(1)IgG closely match the B-cell gene cluster that Schmidt et al. find to be prognostic of increased survival among highly proliferating tumor samples[79]. Contrary to our results, and to that of Rody et al., for tumor samples stratified by proliferation, Schmidt et al. fail to find any association between survival and the expression of their T-cell gene cluster. Rody et al. attribute this discrepancy to possible differences in cohorts and/or treatments, but the difference in results may also be due to the composition of the respective T-cell gene clusters or sets. Schmidt et al.'s T-cell cluster contains our immune(1)T-cell genes as a proper subset, but it also contains genes that belong to immune(4)STAT1 and immune(7)complement, neither of which we find to be significantly associated with survival. Hence, the significance of the T-cell genes as a predictor of survival may be attenuated by genes from other sets.

Immediately relevant to the question of T-cell genes versus IgG genes as predicators of survival, Calabro et al., assembled eighteen genes from the literature to measure the presence of lymphocytic infiltrate [80]. They found that this list is somewhat associated with diminished survival time for estrogen-positive samples, but is strongly prognostic of increased survival for estrogen-negative samples. The genes in this list range from CCL5, CD37, CDE3...to IGHG3 and IGJ, which effectively merges our immune(0)T-cell and Immune(1)IgG sets. Therefore, the separate positive associations with survival between immune(0)T-cell and immune(1)IgG for estrogen-negative samples in the Uppsala and NKI datasets appear to confirm Calibro et al.'s results.

The positive effect of the genes in immune(0)T-cell on survival may in part explain the performance of Finak et al.'s stromal-derived classifier [81]. In that study, hierarchical clustering applied to individually matched tumor and normal stroma yielded three clusters of samples that are starkly distinguished by outcome. From the genes that best discriminate between pairs of these clusters, Finak et al. construct a 23-gene classifier. Ten of these 23 are associated with the good outcome cluster of samples. Of these ten, eight belong to the immune(0)T-cell set: GZMA, CD8A, CD52, CD247, CD48, PLEK, RUNX3, and GIMAP5. This suggests that the stromal-derived classifier, in assigning samples to the good, poor, and mixed groups, may be powered substantially by the association between T-cell genes and increased survival. If this is the case, then the stromal-derived classifier provides yet more evidence for the association between T-cell genes and survival for estrogen-negative breast cancer observed in the Uppsala and NKI data.

The capacity of Teschendorff et al.'s seven gene immune response module to identify good outcome samples from among estrogen-negative tumors may constitute yet more support for this association. Those seven genes span at least four of our immune sets with immuned(0)T-cell represented by LY9, immune(1)IgG by IGLC2 and TNFRSF17, immune(2)MHC-I by HLA-F, and immune(7)complement by C1QA. It is interesting to note that in the attempt to validate this seven-gene module on independent data, only four of the seven genes prove significant, and of these, three belong to either immune(0)T-cell (LY9), or to immune(1)IgG (IGLC2 and TNFRSF17). If these are the genes that are driving the performance of the Teschendorff et al. immune response module, then the effectiveness of that signature for predicting increased survival among estrogen-negative samples is consistent with the immune(0)T-cell and immune(1)IgG survival results for estrogen-negative samples in the Uppsala and NKI data. Overall, the strong association between the T-cell gene set and survival, and the milder association between the immunogloblulin/B-cell gene set and survival, as identified in the enumerations, appears to converge with the important results of each of these several studies despite large differences in approach and research design.

#### Immune(3)/interferon

The immune(3)/interferon set closely resembles the cluster of "interferon response" genes identified by Buess et al. [82]. In that experiment fibroblasts were co-cultured with several breast cancer cell lines to investigate cell-cell signalling between stroma and malignant epithelial cells. In comparing the gene expression of co-cultures to matched monocultures, the starkest difference involved interferon-related genes which were preferentially expressed in co-cultures of fibroblasts with estrogen negative cell lines. Of the twelve genes in the immune(3) gene set, as realized for example in the NKI data, five belong to this "interferon response": *OAS1, OAS2, MX1, MX2*, and *IFIT1*. The remaining seven include: *IFIT4, ISG15, OS4, MTAP4A, USP18, G1P3*, and *GS3686*. Buess et al show that these interferon genes are significantly associated with shorter survival time in the NKI data. The enumeration of the NKI295 confirms this finding.

### The stromal gene sets factor stromal signaling

Efforts to delineate the interaction between stromal cells and epithelial tumor are challenged by the complexity of the microenvironment, which has been defined as all components of the mammary gland other than luminal and/or tumor epithelial cells [83]. The effective division of the stroma-related genes into six gene sets by the enumerations may be relevant to this problem. Unlike the immune sets which appear to be distinct, monolithic entitites, the stromal sets resemble a constellation with a core entity, which we designate "stromal(0)", accompanied by several satellite sets, "stromal(1), stromal(2), stromal(3) and stromal(5). Each of these is detected as a self-contained and independent set under at least one combination of partition "size" and "tolerance" parameters, but, as the algorithm increases the partition size, and relaxes the stringency of what qualifies as a match, these sets tend to quickly merge into an omnibus stromal set. Despite this, the importance of distinguishing the smaller sets becomes apparent in the survival analysis: five of the six stromal gene sets are associated with increased survival, several exceptionally so with log-rank χ^2 ^values in excess of 20.

#### Stromal(0) = core DTF stromal signature

The stromal(0) set, induced in each of the four enumerations, is composed of many, if not all of the collagen and ECM remodelling enzymes featured in West et al's desmoids-type fibromatosis stromal signature (DTF) [84-86]. Prominent genes include: *SPARC, CSPG2, FBLN2, FBN1*, and type-I, type-III, and type-VI collagen genes. That signature was devised as a proof of concept for a larger, on-going program that exploits the mono-cellular property of soft tissue tumors to inductively define subtypes (or states) of fibroblastic stroma cells [84,85,87]. The original DTF signature, comprised of genes differentially expressed between two types of soft tissue tumors, was refined for the purpose of identifying a distinctive stromal response in breast cancer. In five datasets, including three of the four used in this paper, the DTF stromal response identifies a subset of breast cancer patients who experience increased survival. The two versions of the DTF signature contain 182 and 66 genes, respectively. In an analysis of the functional relations among the proteins that correspond to these genes, this list is reduced further to a protein-protein network of 20 genes [86]. The close relationship between the DTF signature and the stromal(0) gene set as realized in each of the four data sets can be conveyed with respect to these twenty essential genes (Additional File 7).

#### Stromal(1)/COL11A1

Among the six stromal sets, stromal(1)/COL11A1 is the only one that is negatively related to survival, though this is apparent only when inspecting the entire family of sets detected. In the enumerations, this gene set, consisting exclusively of *COL11A1 *and *FN1 *probe sets, is almost always subsumed in a larger stromal set composed essentially of stromal(0)/DTF genes. Nevertheless it appears to be a distinct entity under some of the parameter combinations that control the stringency of the match. It is interesting to observe that the survival value of the stromal(0) set, as measured by log-rank, and which is generally positive, abruptly goes to zero as COLL11A1 merges with that set. This scenario is played out in all four datasets. The implication is that important relationships between stromal expression and outcome can be lost unless the stromal genes are decomposed into their constituent sets.

#### Stromal(2)/LAMA2

The stromal(2) gene set is detected in the three Affymetrix data sets and is characterized by *LAMA2 *and *COL14A1*. The genes in this gene set tend to merge with the genes in stromal(3)/DARC as the size of the partition is increased and the stringency of the match is relaxed. Stromal(2)/LAMA2 is significantly associated with increased survival: χ^2 ^13.94 (p = 0.0001) for the Uppsala251, χ^2 ^20.01, p = 0.000007 for Stockholm159, and χ^2 ^3.57, p = 0.05 for TRANSBIG198.

#### Stromal(3)/DARC

The stromal(3)/DARC gene set is detected in the Uppsala and Stockholm cohorts and is positively associated with survival in both: χ^2 ^6.43 (p = 0.01) for the Uppsala251, χ^2 ^13.52 (p = 0.0002) for the Stockholm159. Genes in this set include: *DARC, TNXB, CCL14, LDB2, LHFP*, and *C7*. Duffy antigen receptor for chemokines (*DARC*) is believed to suppress tumor metastasis through two mechanisms: by sequestering angiogenic chemokines [88,89] and by inducing KAI1/CD82 tetraspandin in tumor cells, thereby causing senescence [90-92].

The merozoite of *P. vivax *malaria enters red blood cells via DARC, consequently individuals who lack DARC are resistant to that strain of malaria[93]. The association between the lack of expression of DARC in a large percentage of black men and increased rates of aggressive prostate cancer, compared to whites, has been established [94]. A similar relationship appears to hold between black women and the increased incidence of more aggressive breast cancer. It is estimated that 70% of blacks of West African descent lack the expression of DARC, which is a population that suffers high rates of both prostate and breast cancer [91,95]. A second gene in the stromal(3) gene set, tenascin B (*TNXB*), is down-regulated during the tumor progression of neurofibromatosis [96].

#### Stromal(4)/ADAMTS5

Among the smallest of the stromal sets, Stromal(4) is comprised of *ADAMTS5*, *ZNF288 *(*ZBTB20*), and four Agilent probes that lack gene names. It is significantly associated with increased survival in the NKI295 data: χ^2 ^3.8, p = 0.05, when partitioned at the median, χ^2 ^8.63, p = 0.003, when partitioned by first and last quartiles.

#### Stromal(5)/decorin

Stromal(5) consists almost exclusively of DCN and FBLN1 probe sets. Like the other small stromal sets, with the exception of stromal(4)/ADAMTS5, this set tends to merge with stromal(0), but conceptually and empirically it should be treated as a distinct entity. While stromal(0) is virtually defined by the DTF signature[85] neither *DCN *nor *FBLN1 *are found among the 493 genes in the original list of genes that discriminate between DTF and SFT [84]. In contrast, DCN and FBLN1 are both prominent on the list of myoepihelial genes identified by Allinen et al. in a SAGE-based sequential purification of cell types [97]. Therefore, stromal(5) might better be labelled "myoepithelial". In keeping with reports of the positive effect of myoepithelial cells in co-cluture experiments [98], stromal(5)/decorin is highly significant in all four of the datasets, though in the NKI295 it is apparent that it merges with genes from stromal(0). This may be partially explained by the fact that DCN is spotted only once on the Agilent chip, while it is represented by four probe sets on the Affymetrix U133a. As an experiment, if we used the single Agilent decorin probe as a surrogate for stromal(5). Defined in this way the stromal(5)/decorin gene set proves exceptionally significant as a prognosticator of increased survival in the NKI295 (χ^2 ^15.7, p = 7.51e-05). Across the four datasets the substantive finding is that stromal(5), principally decorin, appears to be strongly associated with increased survival for patients at risk of early onset metastasis.

In sum, of the six stromal gene sets, five are significantly associated with increased survival in one or more of the data sets. This appears to be further evidence of the normalizing effect of stroma. Coculture/coinjection experiments have convincingly shown that aberrant stromal cells are required to promote tumor formation in epithelial cells, and the reverse has also been shown, namely that tumorigenic epithelial cells can revert to normal in the presence of normal stroma [98-100]). Decomposing stromal signalling into its constituent gene sets (and the mechanisms they reflect) may contribute to an understanding of the complex of cell types and signals that comprise the mircroenvironment which is an active participant in the initiation and progression of cancer [3,101,102].

## Conclusions

A research program of hierarchical clustering and data visualization has proven immensely productive for more than a decade [103,104]. But continued reliance on this research script dependent on hierarchical clustering may inhibit the further exploration of large genomic data sets. Here we offer an alternative program for the unsupervised exploration of microarray data, one that delivers all that the standard script delivers plus considerably more.

The paper is merely a proof of the concept that an enumeration of all the genes-by-samples sets in breast cancer is computationally feasible and substantively useful. By enumerating the gene sets in three data sets that use the industry standard Affymetrix U133a, we identify gene sets that are conserved across experiments on a single platform. By enumerating the gene sets in a forth dataset, the NKI data spotted on a custom Agilent chip, we identify gene sets that are conserved across platforms. In terms of substantive results, nearly 40% of the sets detected prove to be significantly associated with survival. These include subsets of immune response and stromal signalling genes involved in the complex interactions between the epithelial tumor and its microenvironment.

From the first microarray-based studies of cancer, a fundamental challenge has been that of data reduction. The task is to filter and factor these large matrices such that statistical modelling is possible. Over the course of a decade, expression-based analysis has progressed from single-factor designs, e.g., regressing groups (subtypes) on survival [9], to two-factor designs, as exemplified by studies that first stratify on estrogen status (or HER2 status, or proliferation), then proceed to identify sets or modules of immune genes that correlate with survival [79,80,105,106]. Using the gene sets induced by the enumerations, at this point the stage may be set for exploring the effects and interactions of multiple sets of genes as determinants of the progress and outcome of disease. These sets, or more specifically the partitions they induce, can be conveniently incorporated into (survival) decision trees. In this way, the best results to date regarding, for example, estrogen status and immune response, can be further qualified and extended via-a-vis important additional factors, such as stromal-status.

The rank-based matching algorithm (and the grid search scheme that generates the enumeration), can be applied to any matrix of expression values. As such, it should be useful for the exploration of a broad range of biological and medical data. Limitations of the algorithm are addressed in Additional File 8. Describing the method as a "direct" approach is a direct reference to Hartigan's original work with two-way clustering [14]. Input is a matrix of real numbers; output is a set of submatrices that can be read off the original data matrix (with rows and columns appropriately permuted). Because it works with the entire data set, it dispenses with the standard data reduction steps, e.g., restricting analysis to "differential" genes. Multiple testing is effectively controlled by classical counting rules. The submatrices (gene sets) discovered can be immediately interpreted using the original variables, namely, in the present context of breast cancer, in terms of genes and tissue samples. Because the clusters are "two-way", they reveal an association between possible biological mechanisms embodied in the gene sets and subsets or subclasses of breast cancer.

## Authors' contributions

DM is responsible for concept and original implementation. DM and JM wrote the paper. Both authors read and approved the final manuscript.

## Supplementary Material

Additional file 1 **Gene sets significant for survival in the Uppsala data set**. Click here for file

Additional file 2 **35 gene sets detected in the Uppsala (Miller 2005) data set**. Click here for file

Additional file 3 **31 gene sets detected in the Stockholm (Pawitan 2005) data set**. Click here for file

Additional file 4 **38 gene sets detected in the TRANSBIG (Desmedt 2007) data set**. Click here for file

Additional file 5 **35 gene sets detected in the NKI (van de Vijver 2002) data set**. Click here for file

Additional file 6 **Seven immune gene sets mapped to seven immune metagenes (Rody 2009)**. Click here for file

Additional file 7 **Stromal(0) genes mapped to DTF genes (West 2005; Beck 2008)**. Click here for file

Additional file 8 **Limitations of the enumerations as implemented**. Click here for file
