# Supplementary material for: Enumerating the gene sets in breast cancer, a "direct" alternative to hierarchical clustering
Source: BMC Genomics. 2010 Aug 23;11:482. doi: 10.1186/1471-2164-11-482 (PMC2996978; doi:10.1186/1471-2164-11-482)
Supplement: Additional file 1 — Gene sets significant for survival in the Uppsala data set. [file 1471-2164-11-482-S1.DOC]

| **10 gene sets prognostic of increased survival in Uppsala cohort** | | | | | | | | | | | | | | | | | | | | | | |  | | | | |
| --- | --- | --- | --- | --- | --- | --- | --- | --- | --- | --- | --- | --- | --- | --- | --- | --- | --- | --- | --- | --- | --- | --- | --- | --- | --- | --- | --- |
| **stromal(0)** | FBN1 CDH11 CDH11 CSPG2 CSPG2 AEBP1 FBN1 THBS2 COL5A1 CSPG2 CSPG2 COL5A1 | | | | | | | | | | | | | | | | | | | | | |  | | | | |
|  | COL5A1 ADAM12 COL5A2 CSPG2 ADAM12 SPON1 FAP LRRC15 ASPN COL5A2 MXRA5 | | | | | | | | | | | | | | | | | | | | | |  | | | | |
|  | SPARC SPON1 GLT8D2 HTRA1 CTSK COL10A1 COL10A1 DCN DCN DCN COL6A2 SPARC | | | | | | | | | | | | | | | | | | | | | |  | | | | |
|  | COL1A2 COL3A1 COL1A1 COL1A2 COL6A1 COL3A1 DCN 211161_s_at COL6A3 COL1A1 | | | | | | | | | | | | | | | | | | | | | |  | | | | |
| **stromal(2)** | LAMA2 IGF1 IGF1 C10orf56 C10orf56 MFAP4 COL14A1 LAMA2 ZNF423 ABCA8 | | | | | | | | | | | | | | | | | | | | | |  | | | | |
| **stromal(3)** | CAV1 CAV1 GNG11 208944_at LHFP LDB2 | | | |  | | | |  | | | | |  | | | | | |  | | | | | | |  |
| **stromal(5)** | DCN FBLN1 FBLN1 DCN DCN DCN | | | |  | | | |  | | | | |  | | | | | |  | | | | | | |  |
| **adipose** | FABP4 PLIN ADIPOQ GPD1 RBP4 LPL LPL G0S2 ADH1B ADH1B | | | | | | | | | | |  | | | | | |  | | | | | | |  | | |
| **immune(5)** | HLA-DRB4 HLA-DRA HLA-DRB1 CD74 HLA-DRA HLA-DPA1 HLA-DRB1 HLA-DMB HLA-DMA | | | | | | | | | | | | | | | | | | | | | |  | | | | |
|  | HLA-DRB5 HLA-DPB1 |  | |  | | |  | | | |  | | | | |  | | | | | |  | | | | | |
| **immune(6**) | SERPING1 ANXA1 SRPX C1S C1R CFH CUGBP2 SERPINF1 | | | | | | | | | | |  | | | | | |  | | | | | | |  | | |
| **TPSAB1** | TPSAB1 TPSB2 TPSAB1 TPSAB1 TPSAB1 TPSAB1 TPSA | | | | | | |  | | | | |  | | | | | |  | | | | | | |  | |
| **7 gene sets prognostic of decreased survival in Uppsala cohort** | | | | | | | | | | | | | | | | | | | | | | |  | | | | |
| **histone** | HIST1H2BF HIST1H2BE HIST1H2BH H2BFS HIST1H2BK HIST1H2BD | | | | | | | | | | | | | | | |  | | | | | | |  | | | |
| **GAPDH** | SLC25A3 HSP90AB1 PTGES3 SET YWHAQ PGAM1 ATP5B UQCRH ATP5G3 PDIA6 XRCC5 | | | | | | | | | | | | | | | | | | | | | |  | | | | |
|  | SET GAPDH C19orf10 PDIA6 GAPDH AFFX-HUMGAPDH/M33197_3_at | | | | | | | | | | | | | | | |  | | | | | | |  | | | |
|  | AFFX-HUMGAPDH/M33197_5_at AFFX-HUMGAPDH/M33197_M_at HINT1 SLC25A5 UBE2D3 | | | | | | | | | | | | | | | | | | | | | |  | | | | |
|  | PGK1 PGK1 RAN RPS10 ATP5O CCT4 PSMD1 YME1L1 PRDX4 EIF1 SDHB NDUFS1 | | | | | | | | | | | | | | | | | | | | | |  | | | | |
|  | LOC56902 COX5A EEF1E1 UGP2 CAND1 ATP5G3 PDIA6 HINT1 RAB1A 208799_at | | | | | | | | | | | | | | | | | | | | | |  | | | | |
|  | HINT1 UQCRFS1 TMEM4 PCMT1 RPS10 KPNA2 EIF1 EIF1 MAPK1 OAZ1 YME1L1 CAB39 | | | | | | | | | | | | | | | | | | | | | |  | | | | |
|  | COPS4 MCTS1 MORF4 SET | |  | | |  | | | |  | | | | |  | | | | | |  | | | | | | |
| **CD24** | CD24 CD24 CD24 CD24 CD24 CD24 | | | |  | | | |  | | | | |  | | | | | |  | | | | | | |  |
| **AFFX-M27830_5** | AFFX-HUMRGE/M10098_3_at AFFX-HUMRGE/M10098_5_at AFFX-HUMRGE/M10098_M_at | | | | | | | | | | | | | | | | | | | | | |  | | | | |
|  | AFFX-r2-Hs18SrRNA-3_s_at AFFX-r2-Hs18SrRNA-5_at AFFX-r2-Hs18SrRNA-M_x_at | | | | | | | | | | | | | | | | | | | | | |  | | | | |
|  | AFFX-r2-Hs28SrRNA-3_at AFFX-r2-Hs28SrRNA-M_at AFFX-M27830_5_at | | | | | | | | | | | | | | | |  | | | | | | |  | | | |
|  | AFFX-M27830_M_at |  | |  | | |  | | | |  | | | | |  | | | | | |  | | | | | |
| **GNAS** | GNAS GNAS GNAS GNAS GNAS GNAS | | | |  | | | |  | | | | |  | | | | | |  | | | | | | |  |
| **16q13** | MT1H MT1X LOC645745 MT2A MT1F MT1E MT1M 204326_x_at MT1F | | | | | | | | | | | | | | | |  | | | | | | |  | | | |
| **proliferation** | CCNB2 OAS1 UBE2C DLG7 MELK CENPA CENPF KIF2C BUB1 TPX2 CEP55 BIRC5 TTK | | | | | | | | | | | | | | | | | | | | | |  | | | | |
|  | CDKN3 DKFZp762E1312 AURKA AURKA FOXM1 PTTG1 MAD2L1 ESPL1 KIF4A LOC146909 | | | | | | | | | | | | | | | | | | | | | |  | | | | |
|  | RACGAP1 ESPL1 KIFC1 KIF11 PRC1 ASPM CCNB1 BUB1B CDC2 CDC2 NEK2 NUSAP1 | | | | | | | | | | | | | | | | | | | | | |  | | | | |
|  | CDC2 KIAA0101 TOP2A KIF20A HMMR RRM2 RRM2 TACC3 ZWINT | | | | | | | | | | |  | | | | | |  | | | | | | |  | | |
