# Supplementary material for: Enumerating the gene sets in breast cancer, a "direct" alternative to hierarchical clustering
Source: BMC Genomics. 2010 Aug 23;11:482. doi: 10.1186/1471-2164-11-482 (PMC2996978; doi:10.1186/1471-2164-11-482)
Supplement: Additional file 2 — 35 gene sets detected in the Uppsala (Miller 2005) data set. [file 1471-2164-11-482-S2.DOC]

35 Miller 2005 core sets

**16q13** MT1H MT1X LOC645745 MT2A MT1F MT1E MT1M 204326_x_at MT1F

**adipose** FABP4 PLIN ADIPOQ GPD1 RBP4 LPL LPL G0S2 ADH1B ADH1B

**AFFX-BioC-5_at** AFFX-BioB-3_at AFFX-BioDn-3_at AFFX-CreX-3_at AFFX-CreX-5_at

AFFX-r2-Ec-bioB-5_at AFFX-r2-Ec-bioD-5_at AFFX-r2-P1-cre-3_at

AFFX-r2-P1-cre-5_at AFFX-r2-Ec-bioC-5_at AFFX-r2-Ec-bioC-3_at

AFFX-BioC-3_at AFFX-r2-Ec-bioB-M_at AFFX-r2-Ec-bioD-3_at AFFX-BioDn-5_at

AFFX-r2-Ec-bioB-3_at AFFX-BioC-5_at

**AFFX-M27830_5** AFFX-HUMRGE/M10098_3_at AFFX-HUMRGE/M10098_5_at AFFX-HUMRGE/M10098_M_at

AFFX-r2-Hs18SrRNA-3_s_at AFFX-r2-Hs18SrRNA-5_at AFFX-r2-Hs18SrRNA-M_x_at

AFFX-r2-Hs28SrRNA-3_at AFFX-r2-Hs28SrRNA-M_at AFFX-M27830_5_at

AFFX-M27830_M_at

**ACTG1** ACTG1 ACTG1 ACTG1 ACTG1 ACTG1 ACTG1 ACTG1 ACTG1

**basal** TRIM29 KRT5 DST KRT17 KRT14 TP73L KRT17 KRT6B

**CD24** CD24 CD24 CD24 CD24 CD24 CD24

**ERBB2** STARD3 GRB7 ERBB2 ERBB2 PERLD1 PERLD1 PPARBP CRKRS

**estrogen** CA12 CA12 ESR1 GATA3 GATA3 GATA3 CA12 JMJD2B JMJD2B JMJD2B CA12 CA12 TBC1D9

TBC1D9 FOXA1 SLC7A8

**GAPDH** SLC25A3 HSP90AB1 PTGES3 SET YWHAQ PGAM1 ATP5B UQCRH ATP5G3 PDIA6 XRCC5 SET GAPDH

C19orf10 PDIA6 GAPDH AFFX-HUMGAPDH/M33197_3_at AFFX-HUMGAPDH/M33197_5_at

AFFX-HUMGAPDH/M33197_M_at HINT1 SLC25A5 UBE2D3 PGK1 PGK1 RAN RPS10 ATP5O CCT4

PSMD1 YME1L1 PRDX4 EIF1 SDHB NDUFS1 LOC56902 COX5A EEF1E1 UGP2 CAND1 ATP5G3 PDIA6

HINT1 RAB1A 208799_at HINT1 UQCRFS1 TMEM4 PCMT1 RPS10 KPNA2 EIF1 EIF1 MAPK1 OAZ1

YME1L1 CAB39 COPS4 MCTS1 MORF4 SET

**GGT1** GGT1 208284_x_at GGT1 GGTLA4 GGT1 GGT2

**GNAS** GNAS GNAS GNAS GNAS GNAS GNAS

**hemoglobin** HBA1 HBB HBA1 HBB HBA1 HBA2 HBA2 HBB HBA2

**histone**  HIST1H2BF HIST1H2BE HIST1H2BH H2BFS HIST1H2BK HIST1H2BD

**immune(0)** CD48 CD37 LCK IL10RA CD2 TNFRSF7 GZMK PTPRC CORO1A TRAC PRKCB1 SH2D1A 210915_x_at 210972_x_at 211796_s_at 213193_x_at CD3D CD52 ARHGAP25 CCL5 SPOCK2 IL2RG CCL5 CD52

GZMA CD247 EVI2B PTPRC CD53 KLRK1 IRF8 LTB CXCL9

**immune(1)** 211645_x_at 215176_x_at 216207_x_at 216401_x_at 216576_x_at 217157_x_at

217378_x_at 217480_x_at POU2AF1 209138_x_at 211633_x_at 211634_x_at 211635_x_at 211637_x_at 211641_x_at 211643_x_at 211644_x_at 211650_x_at IGLJ3 211868_x_at IGLJ3 211908_x_at 214669_x_at IGLJ3 214777_at 214836_x_at 214916_x_at 214973_x_at

215121_x_at 215379_x_at CTA-246H3.1 216491_x_at 216510_x_at 216557_x_at IGL@

216984_x_at 217148_x_at 217281_x_at 214768_x_at 216365_x_at IGHM 217179_x_at

217236_x_at 221651_x_at 221671_x_at

**immune(2)** HLA-F HLA-B HLA-G HLA-G HLA-B HLA-G HLA-F HLA-C HLA-G HLA-C

**immune(3)** POP4 203153_at IFI44L IFIT3 ISG15 IFI44 OAS3 FLJ20035 USP18

**immune(4)** STAT1 CXCL10 STAT1 CXCL11 CXCL11 AFFX-HUMISGF3A/M97935_3_at

AFFX-HUMISGF3A/M97935_MA_at

**immune(5)** HLA-DRB4 HLA-DRA HLA-DRB1 CD74 HLA-DRA HLA-DPA1 HLA-DRB1 HLA-DMB HLA-DMA

HLA-DRB5 HLA-DPB1

**immune(6**) SERPING1 ANXA1 SRPX C1S C1R CFH CUGBP2 SERPINF1

**immune(7)** IFI30 LAPTM5 DHPS C1QB CD4 FCER1G C1QA TYROBP LAPTM5 SLC15A3 MS4A6A LY86

**LST1** LST1 LST1 LST1 LST1 LST1 LST1

**NFIB** NFIB NFIB NFIB NFIB NFIB NFIB

**PPP1R12A** KIAA0100 SS18 SRRM2 UBN1 SFRS2IP SFRS2IP

**proliferation** CCNB2 OAS1 UBE2C DLG7 MELK CENPA CENPF KIF2C BUB1 TPX2 CEP55 BIRC5 TTK CDKN3

DKFZp762E1312 AURKA AURKA FOXM1 PTTG1 MAD2L1 ESPL1 KIF4A LOC146909 RACGAP1 ESPL1

KIFC1 KIF11 PRC1 ASPM CCNB1 BUB1B CDC2 CDC2 NEK2 NUSAP1 CDC2 KIAA0101 TOP2A KIF20A

HMMR RRM2 RRM2 TACC3 ZWINT

**ribosomal(0)** RPL37A EEF1A1 206559_x_at RPL23A 208834_x_at TPT1 TPT1 RPL23A EEF1A1 EEF1A1

AFFX-hum_alu_at

**ribosomal(1)** RHOA PGK1 PGK1 SYPL1 HSPA8 HSPA8 DAZAP2 PGK1 HSPA8 RAB14 SKP1A RAB1A UQCRC2

SET SET CCT4

**ribosomal(5)** SAMM50 CCT2 NDUFAB1 SKP1A ATXN10 TM2D1 MCTS1 HIRIP5

**stromal(0)** FBN1 CDH11 CDH11 CSPG2 CSPG2 AEBP1 FBN1 THBS2 COL5A1 CSPG2 CSPG2 COL5A1 COL5A1

ADAM12 COL5A2 CSPG2 ADAM12 SPON1 FAP LRRC15 ASPN COL5A2 MXRA5 SPARC SPON1 GLT8D2

HTRA1 CTSK COL10A1 COL10A1 DCN DCN DCN COL6A2 SPARC COL1A2 COL3A1 COL1A1 COL1A2

COL6A1 COL3A1 DCN 211161_s_at COL6A3 COL1A1

**stromal(2)** LAMA2 IGF1 IGF1 C10orf56 C10orf56 MFAP4 COL14A1 LAMA2 ZNF423 ABCA8

**stromal(3)** CAV1 CAV1 GNG11 208944_at LHFP LDB2

**stromal(5)** DCN FBLN1 FBLN1 DCN DCN DCN

**TPSAB1** TPSAB1 TPSB2 TPSAB1 TPSAB1 TPSAB1 TPSAB1 TPSAB1

**UBE2D2** 208246_x_at 210679_x_at ZNF160 LOC152719 216524_x_at 217679_x_at CEP27 SLC35E1

215600_x_at KNS2 207730_x_at
