# Supplementary material for: Enumerating the gene sets in breast cancer, a "direct" alternative to hierarchical clustering
Source: BMC Genomics. 2010 Aug 23;11:482. doi: 10.1186/1471-2164-11-482 (PMC2996978; doi:10.1186/1471-2164-11-482)
Supplement: Additional file 3 — 31 gene sets detected in the Stockholm (Pawitan 2005) data set. [file 1471-2164-11-482-S3.DOC]

31 Pawitan core sets

**16q13** 204326_x_at MT1H MT1X MT2A MT1E MT1M MT1F

**adipose** LPL LPL FABP4 PLIN ADIPOQ RBP4 G0S2 ADH1B ADH1B CHRDL1

**AFFX-BioC-5_at** AFFX-BioB-5_at AFFX-BioC-5_at AFFX-BioDn-3_at AFFX-CreX-5_at

AFFX-r2-Ec-bioC-3_at AFFX-r2-Ec-bioC-5_at AFFX-r2-Ec-bioD-3_at

AFFX-r2-Ec-bioD-5_at AFFX-r2-P1-cre-5_at AFFX-BioDn-5_at AFFX-BioC-3_at

AFFX-CreX-3_at AFFX-r2-Ec-bioB-3_at AFFX-r2-P1-cre-3_at AFFX-r2-Ec-bioB-M_at

**AFFX-M27830_5** AFFX-HUMRGE/M10098_3_at AFFX-HUMRGE/M10098_5_at AFFX-HUMRGE/M10098_M_at

AFFX-r2-Hs18SrRNA-3_s_at AFFX-r2-Hs18SrRNA-5_at AFFX-r2-Hs18SrRNA-M_x_at

AFFX-r2-Hs28SrRNA-5_at AFFX-r2-Hs28SrRNA-M_at

**ACTG1** ACTG1 ACTG1 ACTG1 ACTG1 ACTG1 ACTG1 ACTG1 ACTG1

**basal** KRT5 DST KRT17 MIA KRT14 KRT17

**CD24** CD24 CD24 CD24 CD24 CD24 CD24

**estrogen** CA12 CA12 GATA3 GATA3 GATA3 CA12 TBC1D9 TBC1D9 CA12 CA12 ESR1

**ezrin** DDX3X VAMP3 VAMP3 DAZAP2 AFFX-HSAC07/X00351_5_at AFFX-HSAC07/X00351_M_at GDI2

HNRPC TMED2 TMED2 ARF1 CANX HSPA8 STIP1 ENO1 YIPF5 NONO NONO HSP90AB1 TUBB TUBB

**GAPDH** GAPDH GAPDH GAPDH AFFX-HUMGAPDH/M33197_3_at AFFX-HUMGAPDH/M33197_5_at

AFFX-HUMGAPDH/M33197_M_at

**GGT1** GGT1 208284_x_at GGT1 GGTLA4 GGT1 GGT2

**hemoglobin** HBA1 HBB HBA1 HBB HBA1 HBA2 HBA2 HBB HBA2

**histone** HIST1H2BF HIST1H2BI HIST1H2BE HIST1H2BH H2BFS HIST1H2BD HIST1H2BK

**immune(0)** CD2 TRAC 210915_x_at 211796_s_at 213193_x_at CD3D GZMK CD247 CCL5 CD48 CCL5

LCK CCR7 CD52 CD52 CXCL9

**immune(1)** 209138_x_at 211644_x_at 211645_x_at IGLJ3 211868_x_at IGLJ3 214669_x_at IGLJ3

214973_x_at 215121_x_at 215176_x_at 215379_x_at CTA-246H3.1 216207_x_at 216365_x_at 216401_x_at 216576_x_at 216853_x_at 216984_x_at 217148_x_at 217157_x_at 217235_x_at 217281_x_at 217378_x_at 217480_x_at 211637_x_at 211641_x_at 211643_x_at

211650_x_at 214836_x_at 215949_x_at 216491_x_at 216510_x_at 216557_x_at 217179_x_at 211634_x_at 211635_x_at 214768_x_at 221651_x_at 221671_x_at 214916_x_at 214777_at 213502_x_at

**immune(2)** HLA-F HLA-B HLA-G HLA-G HLA-C HLA-B HLA-G HLA-F HLA-G

**immune(4)** STAT1 CXCL10 STAT1 CXCL11 CXCL11 IFIH1 AFFX-HUMISGF3A/M97935_3_at PSMB9

**immune(5)** HLA-DRB5 HLA-DRB4 HLA-DRA HLA-DRB1 HLA-DRA HLA-DPA1 HLA-DRB1 HLA-DQA1 HLA-DMA

**LST1** LST1 LST1 LST1 LST1 LST1 LST1

**OPHN1** OPHN1 C12orf38 MEFV CDC5L LOC56902 215182_x_at RECK LOC125893 SCD5 220725_x_at

SPINLW1 217052_x_at

**PPP1R12A** PPP1R12A ZNF148 BBX SSH1 RAP2A SLC25A36 ZNF148 SPIN C1orf121 LEREPO4 USP1 SART3

BBX PRPF40A KRAS TMEM5 SRRM2 SMC3 SFRS2IP UBXD2 RBM25 KIAA1033 SFRS2IP KIAA1033

C20orf6 PRRC1 TBL1X SCAMP1 NCOA3 GOLGA2 RBM25 214693_x_at NBPF1

**proliferation** FOXM1 OAS1 UBE2C DLG7 AURKA CENPA AURKA KIF2C BUB1 TPX2 CEP55 ASPM PTTG1

KIF11 KPNA2 BUB1B CCNB2 MAD2L1 MELK PRC1 RRM2 RRM2 CDC2 CDC2 CDC2 CCNB1 NUSAP1

KIAA0101

**ribosomal(0)** RPL37A HUWE1 RPL23A 208834_x_at TPT1 TPT1 RPL23A RPS18 RPS2 EEF1A1 RPS2

AFFX-hum_alu_at

**ribosomal(1)** GDI2 HNRPC TUBB HSPA8 TUBB GANAB STIP1 CALU ENO1

**stromal(0)** MMP2 HTRA1 PCOLCE FBN1 THBS2 COL5A1 CSPG2 CSPG2 COL6A2 DCN SPON1 MXRA5 COL5A1

COL5A1 SPARC 212764_at LRRC15 SPON1 ASPN C20orf39 COL5A2 CSPG2 COL6A1 SPARC LUM

CTSK COL5A2 211161_s_at COL3A1 DCN COL1A1 COL1A2 COL1A2 DCN DCN COL3A1 POSTN GLT8D2 COL6A3 CDH11 COL10A1

**stromal(1)** COL11A1 HSD17B6 COL10A1 COL10A1 CORIN HSD17B6 COL11A1

**stromal(2)** ABCA8 IGF1 IGF1 IGF1 IGF1 C10orf56 C10orf56 MFAP4 LAMA2 LHFP FXYD1 CCL14

206093_x_at TNXB TNXB TNXB LAMA2 OGN COL14A1

**stromal(3)** CCL14 206093_x_at DARC TNXB TNXB TNXB AQP1 C7 FXYD1

**stromal(5)** FBLN1 FBLN1 DCN 212764_at JAM3 213429_at DCN CTSK PDGFRL DCN DCN

**TPSAB1** TPSAB1 TPSB2 TPSAB1 TPSAB1 TPSAB1 TPSAB1 TPSAB1 CPA3

**UBE2D2** PDE4C USP34 ZNF160 215600_x_at LOC152719 216858_x_at 217679_x_at SLC35E1 FLJ42393 CEP27
