# Supplementary material for: Enumerating the gene sets in breast cancer, a "direct" alternative to hierarchical clustering
Source: BMC Genomics. 2010 Aug 23;11:482. doi: 10.1186/1471-2164-11-482 (PMC2996978; doi:10.1186/1471-2164-11-482)
Supplement: Additional file 4 — 38 gene sets detected in the TRANSBIG (Desmedt 2007) data set. [file 1471-2164-11-482-S4.DOC]

Desmedt core sets

**16q13** MT1X LOC645745 MT2A MT1E MT1M MT1F 204326_x_at MT1H

**adipose** LPL LPL FABP4 PLIN ADIPOQ G0S2 GPD1 RBP4 CIDEC

**AFFX-BioC-5_at**  AFFX-BioB-5_at AFFX-BioC-3_at AFFX-BioC-5_at AFFX-BioDn-5_at AFFX-CreX-3_at AFFX-CreX-5_at AFFX-r2-Ec-bioC-3_at AFFX-r2-Ec-bioC-5_at AFFX-r2-P1-cre-5_at

AFFX-BioDn-3_at AFFX-r2-Ec-bioD-3_at AFFX-r2-Ec-bioD-5_at

**ACTG1** ACTG1 ACTG1 ACTG1 ACTG1 ACTG1 ACTG1 ACTG1 ACTG1

**basal** SFRP1 SFRP1 SFRP1 GABRP ROPN1 ELF5 SOX10

**CD24** CD24 CD24 CD24 CD24 CD24 CD24

**CD44** CD44 CD44 CD44 CD44 CD44 CD44

**CFLAR** CFLAR CFLAR CFLAR CFLAR CFLAR CFLAR

**ERBB2** STARD3 PPARBP PPARBP GRB7 ERBB2 CRKRS PERLD1 PERLD1

**estrogen** CA12 CA12 ANXA9 CA12 ANXA9 CA12 CA12 GATA3 GATA3 GATA3 MAPT MAPT MAPT JMJD2B TBC1D9 TBC1D9 DNAJC12 JMJD2B JMJD2B SLC39A6 SLC39A6 ESR1 ABAT ABAT ESR1 SCUBE2 PH-4 C9orf116 C9orf116 CIRBP CIRBP TFF1 XBP1 FBP1 GREB1 MYB C10orf116 NPDC1 SLC44A4 215304_at TFF3 FOXA1 NME3 MLPH RHOB MCCC2 SPDEF SPDEF SPDEF

**FOXA1** EIF5B GOLGA4 RBBP6 ZNF638 RBBP6 MPHOSPH10 ZNF638 SDCCAG1 THOC2 IRAK1BP1 BCLAF1 ATRX SMC3 LARP4 213070_at 200915_x_at CEP350 SLK NIPBL STAG2 ATRX STAG2 TROVE2 USP34 ZNF292 NIPBL NIPBL PHIP KIAA1033 TROVE2 ROCK1 KTN1 CEP350 NIPBL

**GAPDH** GAPDH GAPDH GAPDH AFFX-HUMGAPDH/M33197_3_at AFFX-HUMGAPDH/M33197_5_at

AFFX-HUMGAPDH/M33197_M_at

**GGT1** GGT1 208284_x_at GGT1 GGTLA4 GGT1 GGT2

**HBA1** HBA1 HBB HBA1 HBB HBA1 HBA2 HBA2 HBB HBA2

**histone** HIST1H2BF HIST1H2BI HIST1H2BE HIST1H2BH H2BFS HIST1H2BK

**immune(0)** CD52 LCK LCK CD2 CORO1A TRAC TRA@ CD247 SH2D1A 210915_x_at 210972_x_at ITK 211796_s_at 211902_x_at 213193_x_at CD3D NKG7 XCL1 CD52 CCL5 CCL5 IL10RA GZMK GIMAP5 GIMAP5 GZMA TNFRSF7 CTSS HCLS1 CD53 CD48 SELL PTPRC EVI2B PTPRC CYBB CD37 LPXN

LAPTM5 LAPTM5 FCER1G

**immune(1)** POU2AF1 209138_x_at IGHG3 211633_x_at 211634_x_at 211635_x_at 211637_x_at 211640_x_at

211641_x_at 211643_x_at 211644_x_at 211645_x_at 211650_x_at IGLJ3 211868_x_at IGLJ3 214669_x_at IGLJ3 214768_x_at 214777_at 214836_x_at 214916_x_at

214973_x_at 215121_x_at 215176_x_at 215379_x_at CTA-246H3.1 216207_x_at 216365_x_at 216401_x_at 216491_x_at 216510_x_at 216542_x_at 216557_x_at 216576_x_at 216853_x_at 216984_x_at 217148_x_at 217157_x_at 217179_x_at 217227_x_at 217235_x_at

217258_x_at 217281_x_at 217378_x_at 217480_x_at IGHM 221651_x_at 221671_x_at 215214_at IGL@ 211908_x_at

**immune(2)** HLA-F HLA-B HLA-C 209140_x_at HLA-G HLA-G HLA-C HLA-B HLA-A HLA-C HLA-A HLA-C HLA-G HLA-F

**immune(4)** STAT1 AFFX-HUMISGF3A/M97935_3_at AFFX-HUMISGF3A/M97935_5_at

AFFX-HUMISGF3A/M97935_MA_at AFFX-HUMISGF3A/M97935_MB_at

CXCL9 CXCL10 INDO CXCL11 CXCL11 TAP1 PSMB9 IFIH1

**immune(5)** HLA-DMB HLA-DRB5 HLA-DRB4 HLA-DRA HLA-DRB1 HLA-DRA HLA-DPA1 HLA-DQB1 HLA-DRB1 HLA-DMA

**immune(6)** ANXA1 EVI2A IFI16 LY96 IFI16 CLEC2B MDFIC QKI TRIM22 CFH CFH

**immune(10)** CASP1 PSCDBP CASP1 CASP1 CASP1 CASP1

**myo** ACTA2 MYL9 CNN1 TPM2 TAGLN EFEMP2 EFEMP2 TGFB1I1 GAS1 SNAI2 COPZ2

**NFIB** NFIB NFIB NFIB NFIB NFIB NFIB

**PNN** NPIP LOC440354 FNBP4 214035_x_at NPIP LOC339047 PNN NKTR HNRPDL HNRPD PRPF4B CCNL1 SFPQ

**PPP1R12A** EIF5B GOLGA4 RBBP6 SMC3 ZNF638 USP34 RBBP6 ZNF638 THOC2 BCLAF1 TPR SLK ATRX ATRX

ATRX PKN2 KIAA1033 213070_at RANBP2 CEP350 NIPBL NIPBL NIPBL KIAA1033 ROCK1 SFRS2IP

SMC5 SFRS2IP SENP6 PHF3 LRRC40 JMJD1C

**proliferation** CENPA BUB1 CEP55 KIF4A OAS1 UBE2C DLG7 TTK KIF2C TPX2 MCM10 CDCA8 FOXM1 CCNB2 TRIP13 AURKA MELK AURKA PRC1 PTTG1 CCNB1 RACGAP1 BIRC5 DKFZp762E1312 NUSAP1 CDC2 CDC2 ZWINT CDC2 CDKN3

**ribosomal(0)** RPL34 RPL17 RPL12 RPL13A RPL12 RPS4X RPL3 RPS3A RPL37A RPL41 RPS2 EEF1A1 206559_x_at HUWE1 RPL13A RPL3 RPL3 RPL13A RPL3 RPL17 RPS3A RPL17 TPT1 RPS4X RPL12

RPL3 RPS4L2 RPS2 RPS24 RPS23 RPS18 RPL32

**ribosomal(3)** RPL17 RPL12 RPL13A RPL12 RPS4X RPL3 RPS3A EEF1A1 RPL13A RPL3 RPL3 RPL3 RPL17

RPS3A RPL17 RPS4X RPL12 RPL3

**ribosomal(4)** RPL28 RPS11 RPL32 RPL13A RPL10 RPL13A RPL13A RPL13A RPL18

SMARCA4 ASCC3L1 SPEN HUWE1 ARID1A ZUBR1 ARID1A 218917_s_at BRD4 MED12

**stromal(0)** SPARC COL3A1 COL1A2 COL1A2 211161_s_at SPARC COL5A2 COL5A2 222288_at COL6A3 COL1A1 COL5A1 COL5A1 COL5A1 COL3A1 COL6A1 THBS2 COL6A2 COL1A1 CSPG2 CSPG2 CSPG2 CSPG2 CSPG2

BGN AEBP1 CDH11 CDH11 BGN COL1A1 FBN1 LRRC15 COL10A1 COL10A1 HTRA1 SPON1 NID2

**stromal(1)** COL11A1 FN1 FN1 FN1 FN1 COL11A1

**stromal(2)** LAMA2 LAMA2 OGN SPARCL1 C10orf56 C10orf56 COL14A1 LAMA2

**stromal(5)** DCN PDGFRL DCN DCN DCN GLT8D2

**TCF4**  TCF4 TCF4 212387_at 212764_at TCF4 TCF4 SPARCL1

**TPSAB1** TPSAB1 TPSB2 TPSAB1 TPSAB1 TPSAB1 TPSAB1 TPSAB1

**UBE2D2** ZC3H7B USP34 207730_x_at 208082_x_at ZNF611 208246_x_at 210679_x_at ZNF160 FLJ42393 PRDX2 215600_x_at PPP2CA KNS2 216524_x_at 217679_x_at CEP27 FLJ14346 SLC35E1 FLJ12151

FGFR1 216858_x_at
