# Supplementary material for: Enumerating the gene sets in breast cancer, a "direct" alternative to hierarchical clustering
Source: BMC Genomics. 2010 Aug 23;11:482. doi: 10.1186/1471-2164-11-482 (PMC2996978; doi:10.1186/1471-2164-11-482)
Supplement: Additional file 5 — 35 gene sets detected in the NKI (van de Vijver 2002) data set. [file 1471-2164-11-482-S5.DOC]

NKI core sets

**8p11_12** C8ORF2 KIAA0725 ASH2L N/A LSM1 C8ORF2 N/A FLJ11052 FLJ20353

**AANAT** N/A DKFZp434B217 N/A HUMHOXY1 N/A SLC2A4 AANAT HPD

**ACACBC** ACACB N/A PRO1268 N/A N/A N/A N/A N/A

**adipose** PLIN LIPE RBP4 CIDEA ADH1 ADH2

**ATP12A** N/A N/A N/A N/A N/A N/A N/A N/A N/A N/A ATP12A N/A MGAT5

**basal** UGT8 N/A FZD9 DSC2 N/A LOC56963 GABRP PHGDH N/A TONDU N/A N/A N/A FLJ10173 N/A

FOXC1 N/A N/A N/A N/A CDH3

**BCL2L1** N/A PRO2822 N/A N/A BMP3 FGA N/A N/A N/A SLC12A3 DKFZp762M186 DKFZp547C176

BCL2L1 N/A N/A

**EMX2** EMX2 N/A N/A N/A N/A N/A N/A N/A N/A N/A N/A PRO2435 N/A N/A FLJ10043 GABRA1

**ERBB2**  PSMD3 TCAP ERBB2 GRB7 N/A LOC51242 N/A LEP503

**estrogen**  N/A N/A LIV-1 KIAA0575 N/A N/A N/A KIAA0882 N/A ESR1 GATA3 N/A N/A CA12 CELSR1

NAT1 N/A N/A MYB CEGP1 AGR2 ACADSB HNF3A DKFZP434I114 N/A N/A N/A N/A N/A CDC4L

ERBB4 N/A FLJ20262 JCL-1 JCL-1 N/A P28 N/A NET-6 N/A N/A N/A N/A FBP1 TCEAL1 NPDC1

NPDC1 N/A HSPC195 AR TCEB1L TFF1 TFF3 AKR7A3 XBP1 N/A MYO5C PRLR N/A BCAS1 N/A VAV3 ZFP103 TP53TG1 N/A N/A FLJ10980 N/A N/A D5S346 N/A FAAH TP53TG1 GPCR150 N/A FLJ12538 CGI-52 FLJ10116 GALNT7 FLJ20273 N/A RARA N/A N/A N/A N/A ERBB3 TJP3 TSPAN-1 N/A

N/A TJP3 PIB5PA N/A

**FOXA1** CYP NP220 HDCMA18P HDCMC28P N/A FLJ11186 SDCCAG1 AK000060 KTN1 ZFD25 KIAA0874

MPHOSPH10 SSB N/A N/A MATR3 N/A N/A N/A KIAA0244 CBF2 KIAA0164 N/A SSB N/A N/A N/A FLJ20705 MATR3 CSPG6 KIAA0594 KIAA0117 TLOC1 ZNF267 PTK9 NDUFA5 DKFZP434N126 HRB2 TMF1 USP16 TCTE1L KIAA0372 N/A KIAA0204 HNF3A KIAA1033 N/A N/A N/A N/A N/A CD2AP HYPA FLJ20502 FLJ20585 FLJ20727 N/A P115 ARL5 N/A N/A ZNF146 N/A DEK RP42 N/A HSU15552 N/A N/A FLJ10652 ZRF1 N/A NOP5/NOP58 KIAA1565 CDC10 PNN N/A N/A N/A BDH MBNL MBNL N/A N/A STXBP3 LOC51290 KIAA1451 pcnp DKFZP434P0721 N/A N/A PA TOP2B KIAA0266 N/A ARL5 LOC57187

**H2B_histone** H2BFH H2BFJ H2BFK H2BFL H2BFB H2BFB H2BFA

**H3_histone** H3FT H3FA H3FD H3FH H3FJ H3FI

**immune(0)** LTB CD2 CCR7 N/A PLEK CD53 SCYA5 HEM1 LCK LCP2 PTPRCAP N/A TYMSTR N/A BIN2

TRB@ ADORA2BP LTB FLJ11296 SIRP-b2 CD53 CCR5 CD3D CD3Z GPR9 IL10RA KIAA0275

**immune(1)** IGLL1 IGL@ IGL@ POU2AF1 N/A IGKC PIM2 N/A N/A FLJ20260 IGKV3D-15 N/A N/A

**immune(2)** PSMB9 ABCB2 HLA-B HLA-E HUMMHCW1A HLA-A HLA-A

**immune(3)** ISG15 GS3686 N/A IFIT1 IFIT4 MX1 N/A

**immune(5)** CD53 HEM1 LCP2 N/A LILRB2 BIN2 BTK CD53 HLA-DMB DOCK2

**immune(9)** TNFRSF17 N/A N/A SAGE N/A N/A N/A N/A N/A FGF6

**JAG2** N/A DHPS N/A PTPRS IP6K2 EGFL3 CL683 CALCYON FLJ10669 N/A JAG2

**KRT1** MUC2 PCSK2 DSG1 SCG2 N/A HBP17 KRT1 N/A KRT2A LOR KIAA1449 TPS1 TPSB1 FGG N/A COL2A1

CALB3 LOC56934 FIL1(ZETA) N/A SPINK5 SYT4 BCHE LGALS7 PAD N/A N/A SCG3 XP5 N/A

KRT13 N/A MYOC N/A FGB SCYA27 CALCA LOC51617 KRT10 SGCA N/A N/A CDH19 DSC1 SEZ6L DDC

**MAGEB1** N/A POU2F1 SLC5A3 N/A FLJ10257 N/A MAGEB1 N/A N/A N/A N/A

**MMP17** IDH3G N/A MAGEA9 JMJ MMP17 HSPC210 N/A FLJ20241

**NRGN** C8FW PP32R1 N/A N/A NRGN POU4F1 N/A N/A N/A N/A N/A FLJ10748 N/A DKFZp434I0428

**proliferation** STK12 BUB1 KNSL6 FLJ10468 DKFZp762E1312 N/A KNSL2 CENPA C20ORF1 N/A STK6

PTTG1 STK15 AD024 CCNB2 PTTG2 KIAA0074 RAB6KIFL KIAA0175 N/A ID-GAP UBCH10 PRC1

KNSL5 FLJ10540 LOC51203 PKMYT1

**PTPRO** N/A PTPRO N/A N/A MYBPC3 FLJ20302 GTF2IP1

**RAB18** RAB18 CAPZA2 N/A LOC57213 RAB18 AD022 DLD PSMC6 LOC51714 N/A LOC51068 CCNC

**RAPGEF2** N/A N/A N/A N/A N/A N/A N/A N/A N/A DKFZP586O1422 N/A N/A N/A N/A N/A N/A N/A N/A

N/A N/A N/A N/A N/A N/A N/A N/A N/A N/A N/A N/A N/A LOC51105 N/A N/A N/A N/A N/A

N/A N/A N/A N/A N/A N/A N/A N/A N/A N/A N/A N/A N/A N/A N/A N/A N/A N/A N/A N/A N/A

N/A N/A N/A N/A N/A N/A N/A N/A N/A N/A N/A N/A N/A N/A NSG-X N/A N/A FLJ10619 N/A

N/A N/A N/A N/A N/A N/A N/A N/A N/A N/A N/A N/A N/A N/A N/A N/A N/A N/A N/A N/A N/A

N/A N/A N/A N/A N/A N/A N/A N/A N/A N/A N/A BVES N/A N/A N/A N/A N/A N/A N/A N/A N/A

N/A N/A N/A N/A N/A N/A N/A N/A N/A N/A N/A N/A N/A N/A N/A N/A N/A KIAA0754 N/A N/A

N/A N/A KIAA0007 N/A N/A N/A N/A N/A N/A N/A N/A

**RBM5** N/A PLA2G4B RBM6 RBM5 KIAA1014 N/A N/A NPFF N/A N/A N/A EIF4A2 N/A N/A N/A

**ribosomal(0)** one_barcode RPS2 RPL41 N/A N/A N/A FLJ21794 N/A PRO0628 N/A GDF2 N/A

**ribosomal(2)** OAS1 LTBP4 BAX N/A RPL36 ZFPL1

**SEMG1** N/A N/A N/A N/A SEMG1 SEMG2 N/A N/A N/A N/A N/A N/A

**stromal(0)** SPARC CDH11 THBS2 PRSS11 COL1A2 COL3A1 COL5A2 N/A ADAM12 LOC51339 N/A CRHR1

COL6A3 N/A FBN1 N/A N/A COL6A1 DKFZP564I1922 NID2 N/A LOXL1 FN1

**stromal(4)** N/A ADAMTS5 N/A ZNF288 N/A N/A

**stromal(5)** SPARC PRSS11 CRHR1 N/A N/A LOC51226 N/A COL1A2 COL3A1 SPON1 COL6A1 COL6A3 N/A

FBN1 DKFZP564I1922 CDH11 NID2 COL5A2 N/A C1R NDN CDH5 TGFB3 THBS2 PCOLCE KIAA1474

PDGFRB SERPINF1 DCN FBLN2 N/A RECK PRKCM ADAM12 KIAA1474 CSPG2 SCGF N/A AKAP12

FAP N/A N/A FLRT2 N/A N/A N/A DLC1 N/A PDGFRA ADAMTS2 GARP LOXL1 N/A N/A EMILIN SLUG

N/A MMP14 SRPUL OSF-2 FBLN1 N/A N/A P37NB N/A N/A N/A N/A N/A COL5A3 PTRF N/A N/A

N/A N/A FLJ10157 LOC51339 DAB2 N/A N/A N/A N/A N/A FLJ20129 N/A N/A FLJ21935

CTSK FN1 AEBP1 BMP1 COL10A1 N/A N/A N/A N/A FMOD DPYSL3 ECM2 N/A N/A N/A INHBA IGFBP4 IGFBP7 LAMA4 N/A
