# Supplementary material for: Enumerating the gene sets in breast cancer, a "direct" alternative to hierarchical clustering
Source: BMC Genomics. 2010 Aug 23;11:482. doi: 10.1186/1471-2164-11-482 (PMC2996978; doi:10.1186/1471-2164-11-482)
Supplement: Additional file 6 — Seven immune gene sets mapped to seven immune metagenes (Rody 2009). [file 1471-2164-11-482-S6.DOC]

| immune(0) | immune(1) | immune(2) | immune(3) | immune(4) | immune(5) | immune(7) |
| --- | --- | --- | --- | --- | --- | --- |
| LCK | IgG | MHC-I | interferon | STAT1 | MHC-II | HCK |
| √ CCL5 | √ POU2AF1 | √ HLA-F | MX1 | √ STAT1 | √ HLA-DPB1 | √ IFI30 |
| √ IL2RG | IGLC2 | √ HLA-B | IFI27 | √ GBP1 | √ PRG1 | √ LAPTM5 |
| √ CD48 | √ IGHM | √ HLA-G | OAS1 | √ TAP1 | √ CTSS | ITGB2 |
| √ SELL | [IGH@](mailto:IGH@) | √ HLA-A | IFIT1 | IRF1 | √ HLA-DMB | √ C1QB |
| √ SCYA5 | IGHG1 | √ HLA-C | G1P3 | √ CXCL9 | √ HLA-DRB1 | SLCO2B1 |
| √ LCK | √ IGHG3 | HLA-J | √ IFI44L | √ PSMB9 | LCP2 | CD163 |
| √ GZMA | LOC388078 |  | √ IFIT3 | √ CXCL10 | √ PTPRC | TYROBP |
| √ IL7R | IGKC |  | OAS2 | √ INDO | √ HLA-DRA | √ FCER1G |
| √ KLRK1 | √ IGLJ3 |  | G1P2 | √ CXCL11 | √ CD74 | SLC7A7 |
| √ CD2 | LOC91316 |  | RSAD2 | IFIH1 | √ HLA-DPA1 | CCR1 |
| STAT4 | LOC440871 |  | √ IFI44 |  | √ HLA-DQA1 | √ TFEC |
| √ TNFRSF7 | [IGL@](mailto:IGL@) |  | √ OAS3 |  | √ HLA-DMA | HCK |
| SLAMF1 | IGHD |  | √ FLJ20035 |  |  | NCF2 |
| √ CCR7 | IGLL1 |  |  |  |  | √ LAIR1 |
| √ GZMK | IGKV1D-13 |  |  |  |  | √ CD86 |
| √ CCR2 | IGL |  |  |  |  | √ C1QA |
| LTB | LOC391427 |  |  |  |  | MS4A4A |
| [√ TRA@](mailto:TRA@) | LOC339562 |  |  |  |  | MNDA |
| √ PRKCB1 |  |  |  |  |  | √ AIF1 |
| CD3Z |  |  |  |  |  | DOCK2 |
| √ SH2D1A |  |  |  |  |  | RNASE6 |
| TRBC1 |  |  |  |  |  | √ MS4A6A |
| √ ITK |  |  |  |  |  | LST1 |
| √ CD3D |  |  |  |  |  | LST1 |
| GIMAP5 |  |  |  |  |  | LST1 |
| PLAC8 |  |  |  |  |  | LST1 |
| √ GIMAP4 |  |  |  |  |  | LST1 |
| √ PRG1 |  |  |  |  |  | LST1 |
| √ HCLS1 |  |  |  |  |  |  |
| INPP5D |  |  |  |  |  |  |
| √ CD53 |  |  |  |  |  |  |
| SLA |  |  |  |  |  |  |
| √ PIK3CD |  |  |  |  |  |  |
| √ IRF8 |  |  |  |  |  |  |
| GMFG |  |  |  |  |  |  |
| √ FGL2 |  |  |  |  |  |  |
| √ IL10RA |  |  |  |  |  |  |
| CXorf9 |  |  |  |  |  |  |
| CSF2RB |  |  |  |  |  |  |
| √ LCP2 |  |  |  |  |  |  |
| √ CORO1A |  |  |  |  |  |  |
| HEM1 |  |  |  |  |  |  |
| SELPLG |  |  |  |  |  |  |
| √ EVI2B |  |  |  |  |  |  |
| √ PTPRC |  |  |  |  |  |  |
| √ RAC2 |  |  |  |  |  |  |
| √ LPXN |  |  |  |  |  |  |
| √ARHGAP15 |  |  |  |  |  |  |
| SAMSN1 |  |  |  |  |  |  |
| KIAA0053 |  |  |  |  |  |  |

First row: immune gene sets. Second row: immune metagenes from Rody et al. 2009. Check marks (√) indicate genes in common.
