# Supplementary material for: Enumerating the gene sets in breast cancer, a "direct" alternative to hierarchical clustering
Source: BMC Genomics. 2010 Aug 23;11:482. doi: 10.1186/1471-2164-11-482 (PMC2996978; doi:10.1186/1471-2164-11-482)
Supplement: Additional file 7 — Stromal(0) genes mapped to DTF genes (West 2005; Beck 2008). [file 1471-2164-11-482-S7.DOC]

| DTF protein | stromal(0) | stromal(0) | stromal(0) | stromal(0) |
| --- | --- | --- | --- | --- |
| network | Uppsala | Stockholm | TRANSBIG | NKI |
| CSPG2 | √ CSPG2 | √ CSPG2 | √ CSPG2 | √ CSPG2 |
| FBLN2 | √ FBLN2 | √ FBLN2 |  | √ FBLN2 |
| FBN1 | √ FBN1 | √ FBN1 | √ FBN1 | √ FBN1 |
| MFAP2 | √ MFAP2 | √ MFAP2 |  |  |
| LOX |  |  | √ LOX |  |
| WISP1 | √ WISP1 |  |  | √ WISP1 |
| BGN | √ BGN | √ BGN | √ BGN | √ BGN |
| COL1A1 | √ COL1A1 | √ COL1A1 | √ COL1A1 | √ COL1A1 |
| COL1A2 | √ COL1A2 | √ COL1A2 | √ COL1A2 | √ COL1A2 |
| COL3A1 | √ COL3A1 | √ COL3A1 | √ COL3A1 | √ COL3A1 |
| FN1 | √ FN1 | √ FN1 | √ FN1 | √ FN1 |
| SPARC | √ SPARC | √ SPARC | √ SPARC | √ SPARC |
| SDC1 |  |  |  |  |
| COL6A1 | √ COL6A1 | √ COL6A1 | √ COL6A1 | √ COL6A1 |
| ADAMTS2 |  |  | √ ADAMTS2 | √ ADAMTS2 |
| COL6A2 | √ COL6A2 | √ COL6A2 | √ COL6A2 | √ COL6A2 |
| CTGF |  |  |  | √ CTGF |
| POSTN | √ POSTN | √ POSTN | √ POSTN |  |
| THBS2 | √ THBS2 | √ THBS2 | √ THBS2 | √ THBS2 |
| COL5A1 | √ COL5A1 | √ COL5A1 | √ COL5A1 |  |

Column 1: twenty genes from a protein network representing West et al.’s desmoids-type fibromatosis stromal signature (DTF). Subsequent columns: genes in the stromal(0) gene set as detected, respectively, in the Uppsala, Stockholm, TRANSBIG, and NKI data sets.
