# Supplementary material for: Enumerating the gene sets in breast cancer, a "direct" alternative to hierarchical clustering
Source: BMC Genomics. 2010 Aug 23;11:482. doi: 10.1186/1471-2164-11-482 (PMC2996978; doi:10.1186/1471-2164-11-482)
Supplement: Additional file 8 — Limitations of the enumerations as implemented. [file 1471-2164-11-482-S8.DOC]

## Three limitations of the rank-based partitioning program as implemented in this study, and a note on how labels were assigned to the gene sets.

## [1] The stop rule halts the grid search before important gene sets have been detected.

The grid of parameter combinations that controls the enumeration consists of a range of values for partition size crossed by a range of tolerances for mismatched samples. Computing the bounds on the partition size is straightforward since it is governed by the probability of a random match given the number of samples (*n*), the number of genes (*p*), and the number of draws (essentially *p*, again). The bounds on the second parameter, namely the quality of the match can also be set by a counting rule, but in datasets with more than 200 samples, the values for *t*, for a fixed probability (e.g., Bernoulli’s moral certainty), often results in very large, diffuse sets of genes. So, as a practical matter, after an initial spiral search revealed interesting gene sets, we chose to set tolerance indirectly by a stop rule intended to prevent specific gene sets from merging (which inevitably occurs as *t* is relaxed). Initially we defined a stop rule on the basis of two known or suspected sets, i.e., “proliferation” and “estrogen”. That is, for each partition size *s*, increment *t* starting at *t*=0 (perfect match) to a *t’* until the proliferation and estrogen gene sets merged. In light of interesting structure that became apparent in the immune response genes, we reformulated the stop rule to prevent an interferon/immune gene set from merging with other immune sets. This worked well for the purpose of factoring immune response, and it also served to find subsets of stromal genes, along with the other gene sets reported in the paper. But it had the unfortunate effect of stopping the search before all admissible gene sets could be induced. When we increased the size of the grid of parameter combinations by relaxing *t* beyond what the stop rule prescribed, we found, along with other sets, a gene set containing *FOS, FOSB*, *JUN* and *JUNB* That set that closely resembles a FOS-JUN gene set termed “early response cluster” by Bertucci et al. [1]. We conclude that it would be worthwhile to run the enumerations on a larger grid without the stop rule, viz., bounded only by the combinatorial rules.

[2] Several alternative partition rules yield better results than K-means.

In the survival analysis, for the purpose of defining groups given a gene set, any partition rule can be plugged in. In the past we have used K-means (k=2). In the effort to be conservative, here we partition at the median column sum value, and, in the effort to detect smaller gene sets, we partition by lowest versus highest quartile. A supervised subsampling based partitioning scheme like Camp et al.’s Xtile [2], sometimes yields larger log-rank values, sometimes much larger. The suggestion would be to replace the current partitioning rule with a variant of Xtile. An alternative would be to plug-in a model-averaging scheme like that of Monti et al.s “ensemble consensus clustering” [3, 4].

## [3] The “shape” of the gene sets and the problem of “chaining”.

In graph theoretic terms, with genes as vertices, the gene sets induced by the algorithm constitute graphs for which every pair of vertices are linked by a path of edges, each of which satisfy the matching rule for the given *s* and *t* for the two genes linked by the edge. This is the formal definition of “gene set”. For many of the gene sets, especially the smallest, all of these paths are of length 1, and hence the graphs are complete. But for other gene sets, a path between two genes in the set may consist of more than one edge. And it can be the case that a subset of genes, which may form a complete graph, are linked to a second such subset via a single edge between a gene in the first subset and one in the second. This is the phenomenon of “chaining” well known from single-linkage clustering. We have experimented with cutsets and edge-deletion schemes to force tighter clusters, but the improvement in the coherence of the gene sets often appears to be minor, and to be outweighed by the added computational cost. In any event, this problem of “elongated” clusters arises only in the largest gene sets.

## [4] Assigning labels to gene sets.

Assigning labels to the gene sets is straightforward in cases such as erbb2/17q12, a set which has figured prominently in microarray-based studies for more than a decade. Labelling a number of the new sets is more problematic, though in many cases the label is obvious on the basis of the constituent genes, for example: “histone” or “hemoglobin”. But some sets resist naming. For these we simply select a prominent gene in the set to serve as a surrogate label. This is the case, for example, with the “ezrin” gene set in the Stockholm data. In an attempt to draw guidance from the literature, we map more than 200 previously published gene clusters and lists onto the sets returned by the enumerations. These lists of genes range from amplicons, e.g., [5] and pathways [6] to classifiers/signatures (“wound”, “hypoxia”, “70-gene”...) and call-outs from heatmap figures, e.g., [1, 7]. A problem that arises in assigning labels to sets is that some of the gene clusters described in the literature span multiple distinct sets as detected in the enumerations. This is especially the case for “immune response”, but also obtains for “stromal” and “ribosomal” genes. All in all, across the four datasets there are six such stromal sets, ten immune sets, and four ribosomal sets. The importance of distinguishing these components as distinct entities becomes apparent in the survival analysis. For example, five of the six stromal gene sets are significantly associated with increased survival, while one is significantly prognostic of poor outcome. When these sets are indiscriminately collected into a single superset of stromal genes, that significance vanishes.

1. Bertucci F, Finetti P, Cervera N, Charafe-Jauffret E, Mamessier E, Adelaide J, Debono S, Houvenaeghel G, Maraninchi D, Viens P *et al*: **Gene expression profiling shows medullary breast cancer is a subgroup of basal breast cancers**. *Cancer Res* 2006, **66**(9):4636-4644.

2. Camp RL, Dolled-Filhart M, Rimm DL: **X-tile: a new bio-informatics tool for biomarker assessment and outcome-based cut-point optimization**. *Clin Cancer Res* 2004, **10**(21):7252-7259.

3. Monti S, Tamayo P, Mesirov J, Golub T: **Consensus clustering: a resampling-based method for class discovery and visualization of gene expression microarray data**. *Machine learning* 2003, **52**(1):91-118.

4. Monti S, Savage K, Kutok J, Feuerhake F, Kurtin P, Mihm M, Wu B, Pasqualucci L, Neuberg D, Aguiar R: **Molecular profiling of diffuse large B-cell lymphoma identifies robust subtypes including one characterized by host inflammatory response**. In*.*, vol. 105: Am Soc Hematology; 2005: 1851-1861.

5. Yao J, Weremowicz S, Feng B, Gentleman RC, Marks JR, Gelman R, Brennan C, Polyak K: **Combined cDNA array comparative genomic hybridization and serial analysis of gene expression analysis of breast tumor progression**. *Cancer Res* 2006, **66**(8):4065-4078.

6. Bild AH, Yao G, Chang JT, Wang Q, Potti A, Chasse D, Joshi MB, Harpole D, Lancaster JM, Berchuck A *et al*: **Oncogenic pathway signatures in human cancers as a guide to targeted therapies**. *Nature* 2006, **439**(7074):353-357.

7. Perou CM, Jeffrey SS, van de Rijn M, Rees CA, Eisen MB, Ross DT, Pergamenschikov A, Williams CF, Zhu SX, Lee JC *et al*: **Distinctive gene expression patterns in human mammary epithelial cells and breast cancers**. *Proc Natl Acad Sci U S A* 1999, **96**(16):9212-9217.
